# Boron-Doped Endohedral Metallofullerenes: Synthesis and Computational Analysis of a Family of Heteroatom-Doped Molecular Carbons

**DOI:** 10.1021/acs.inorgchem.4c05122

**Published:** 2025-01-08

**Authors:** Antonio Moreno-Vicente, Sven Schardt, Paul W. Dunk, Josep M. Poblet, Antonio Rodríguez-Fortea

**Affiliations:** † Departament de Química Física i Inorgànica, 16777 Universitat Rovira i Virgili, c/Marcel·lí Domingo 1, 43007 Tarragona, Spain; ‡ Department of Chemistry, Technische Universität Darmstadt, Alarich-Weiss-Straße 8, 64287 Darmstadt, Germany; § Karlsruhe Institute of Technology, Institute for Chemical Technology and Polymer Chemistry, Engesserstr. 20, 76131 Karlsruhe, Germany; ∥ National High Magnetic Field Laboratory, 7823 Florida State University, 1800 East Paul Dirac Drive, Tallahassee, Florida 32310, United States

## Abstract

Gas-phase synthesis and detection of boron-doped nitride clusterfullerenes and a large variety of monometallofullerenes have been achieved using a pulsed laser vaporization cluster source. Density functional theory (DFT) calculations show that the electronic structures of boron-doped endohedral metallofullerenes differ from those of the pristine all-carbon cages due to the lack of one electron upon boron substitution. For monometallofullerenes, this is likely the main reason for the somewhat different abundance distribution observed for boron-doped with respect to all-carbon cages. Moreover, the three carbon atoms directly bonded to B show the most negative charges in the cage, and consequently, metal atoms are primarily placed nearby boron.

## Graphical abstract

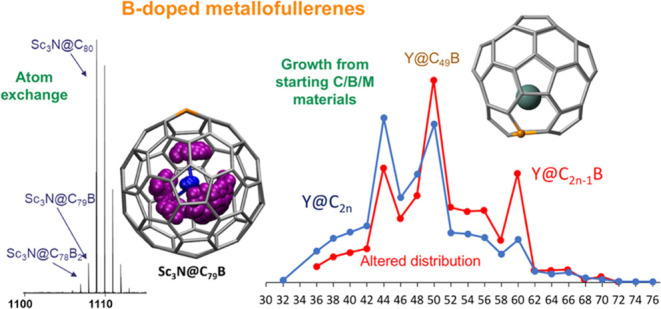

## Introduction

Among the different approaches to modifying fullerenes or other carbon allotropes, one particularly interesting strategy involves substituting atoms within the carbon network to modify their electronic structure and, consequently, their properties. The first heterofullerenes C_60–*x*_B_x_ were already detected a few years after the discovery of C_60_ by laser vaporization of graphite and boron nitride.^1,2^ The preferred elements for substituting carbon atoms of a fullerene are its neighbors in the periodic table, boron and nitrogen, due to their similar size and electronegativity, which make them ideal to form covalent bonds within the cage structure. Boron-doped carbon nanotubes (CNT) show interesting potential as electrocatalysts for the oxygen reduction reaction^3^ as well as increased thermoelectric efficiency compared to undoped single-walled CNT.^4,5^ Fine control of the edges of boron-doped molecular carbons with polycyclic π-skeletons has been recently seen to greatly affect their electronic structure and the photonic properties.^6^ Rational synthetic routes for nitrogen-containing fullerenes were proposed already in the nineties,^7–10^ in addition to production strategies of macroscopic quantities of C_60–*x*_B_*x*_ by arc burning^11–13^ and other techniques.^14,15^ More recently, Kroto and co-workers reported the formation of B-doped fullerenes by direct exposure of pristine C_60_ and C_70_ to boron vapor by means of a pulsed laser vaporization cluster source.^16^ To complement these works, several computational studies on B-doped fullerenes have been reported since the pioneering work of Andreoni et al. on C_59_B.^17^ Disubstituted C_58_B_2_ and systems with higher levels of boron substitution have been thoroughly analyzed.^18–23^ Using the same laser vaporization technique, in a joint experimental and computational work published recently, we have synthesized and detected the first endohedral B-doped fullerenes, U@C_2*n*–1_B (2*n* = 28–60).^24^ The most abundant compound of this family is the smallest uranofullerene, U@C_27_B, the first compound in which uranium can be formally described as highly oxidized U­(VI) with no bond to very electronegative atoms, among other characteristic features. This is one example more of the distinctive host–guest interaction in endohedral metallofullerenes,^25^ a family of fullerenes that were first detected just after the synthesis of C_60_.^26^ Synthesis of the prototypical and first nitride clusterfullerene, Sc_3_N@*I_h_*-C_80_, in 1999 in Dorn’s lab was the beginning of a new period in the field of endohedral fullerenes,^27^ which remains still very active more than 20 years later. The electronic structure of these nitride and other clusterfullerenes can be easily understood by considering a simplified ionic model of interaction. In the case of scandium nitrides (also valid for yttrium, lanthanum, and other lanthanides), there is a formal transfer of six electrons from the cluster to the carbon cage, i.e., (Sc_3_N)^6+^@(*I_h_*-C_80_)^6–^, and many of the properties of the clusterfullerenes can be explained based on their hexa-anions, even though the cluster-cage interaction cannot be considered purely ionic.^28^ Oxide, sulfide and carbide clusterfullerenes of the type M_2_O@C_2*n*_, M_2_S@C_2*n*_ and M_2_C_2_@C_2*n*_ can be seen as (M_2_X)^4+^@(C_2*n*_)^4–^ and many monolanthanofullerenes as M^3+^@C_2*n*_^3–^.^29–35^ Recently, endohedral actinidofullerenes have been synthesized in an electric arc and fully characterized.^36,37^ In monoactinidofullerenes, formal transfer up to four electrons is frequent, but isomer-dependent oxidation state (III or IV) is also observed for U@C_82_.^36–38^ In diactinido or actinido-lanthanido EMFs, the carbon cage acts as a nanocontainer that allows the study of the elusive actinide-actinide or actinide-lanthanide bond,^39–41^ as well as lanthanide-alkaline earth bond.^42^ Carbide and nitride actinide clusterfullerenes have been also characterized.^43–45^ It is remarkable that N-doped metallofullerenes like M_2_@C_79_N or M@C_81_N have been isolated in significant amounts in contrast to B-doped systems.^46–49^

Herein, using a laser vaporization cluster source, we prepare and detect boron-doped nitride clusterfullerenes, Sc_3_N@C_80–*x*_B_*x*_ and Sc_3_N@C_80_B_*x*_, where *x* = 1, 2, and 3, from the pre-existing Sc_3_N@C_80_ exposed to B-containing vapor. Sc_3_N@C_79_B and other members of this family were already studied at computational level several years ago.^50,51^ We also detect a family of B-dopecd endohedral monometallofullerenes M@C_2*n*–1_B (M=Ca, Sr, Sc, Y, La, Pr, Eu, Ho, Ti, Th), which are formed in this case from the boron/carbon starting materials through the bottom-up mechanism.^52^ By means of DFT calculations (BP86/TZP) and CPMD simulations, we characterize the electronic structure and evaluate the different positions in which the boron atoms will be placed for the mono-, di-, and trisubstitution on the nitride clusterfullerenes as well as the behavior of the cluster inside the fullerene cage. We also analyze the electronic structure of B-doped endohedral monometallofullerenes M@C_2*n*–1_B for a widespread set of metals and disclose the differences between their abundance distributions compared to the all-carbon cages M@C_2*n*_.

## Results and Discussion

### Synthesis of Boron-Doped Nitride Clusterfullerenes

Figure 1 shows the mass spectrum obtained by Fourier-transform ion cyclotron resonance (FT-ICR) spectrometry resulting from exposure of pre-existing Sc_3_N@C_80_ to boron vapor. Two groups of important peaks are observed, which show the rich chemistry that is taking place. First, we find around 1100 *m*/*z* a group of peaks that correspond to Sc_3_N@C_80_ and the mono- and disubstituted boron products due to atom exchange. Then, at larger *m*/*z* values, we find the products that incorporate extra boron atoms, which we name growth products. Three groups of peaks, which still show high relative abundance, are referred to the addition of one, two and three boron atoms to the carbon framework, respectively. The peaks for addition of four and five boron atoms are, however, hardly visible.

**Figure 1:**
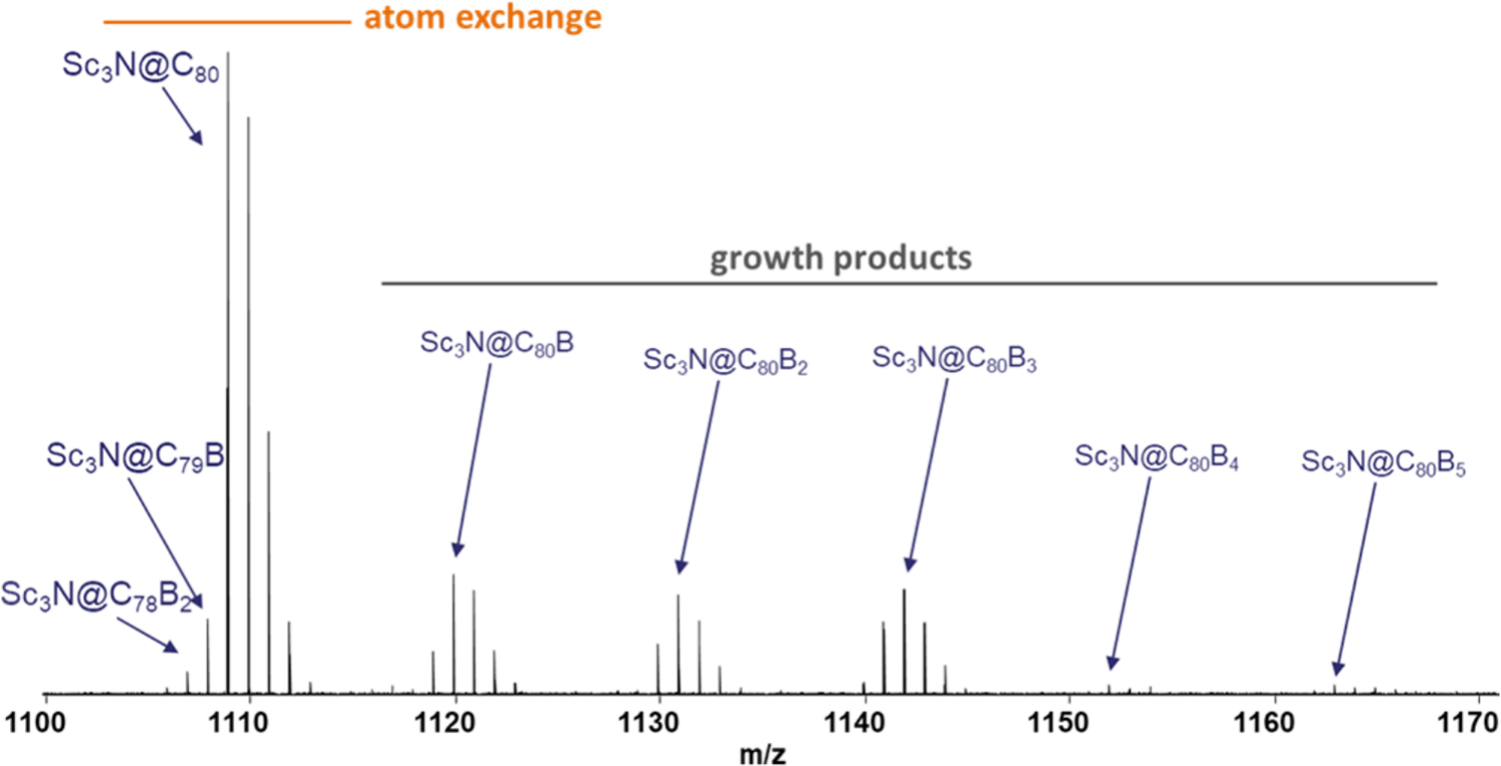
FT-ICR mass spectrum that shows peaks for Sc_3_N@C_80_, the boron-doped clusterfullerene products due to atom exchange, and the addition products at different levels of boron addition.

We here focus on the atom exchange process and analyze in detail Sc_3_N@C_80–*x*_B_*x*_ (*x* = 1–3) systems (see Figure S1). To better understand this atom exchange process, we studied the different positions in which the boron atoms would be placed within the carbon framework, the electronic structure of the boron-doped products and the behavior of the cluster inside the fullerene compared to the all-carbon cage.

### Ionic Model: (Sc_3_N)^6+^@(C_79_B)^6–^

As for the prototypical all-carbon clusterfullerene Sc_3_N@*I_h_* (7)-C_80_, we consider that the main features of the electronic structure of the corresponding B-doped endohedral fullerene are acceptably described by the hexaanionic doped cage (C_79_B)^6–^. Therefore, we have analyzed the substitution of a C atom with a B atom in the pristine *I_h_*-C_80_(7) cage. Due to the high symmetry of this cage, there are only two different positions to substitute C by B, which we name as 665 and 666 depending on whether the substituted carbon is in the center of two hexagon rings and one pentagon or in the center of three hexagons (Figure S3). The energy difference between these two possible hexa-anions C_79_B^6–^ is just 1.5 kcal·mol^–1^, being the 666 isomer slightly more stable without a clear indication of what would be the most favorable position for the substitution in the EMF. Moreover, since they are open-shell systems, the spin density has been analyzed aside from carrying on Bader charge analysis for both structures to evaluate their electronic distribution over the molecule. The spin density is mainly distributed around the position of the boron atom, and it seems slightly more delocalized for the 666 substitution (Figure S4a). It is also remarkable that (Figure 2) the three carbon atoms bonded to the boron are highly negatively charged showing values of around −0.67 in contrast to the value of 1.69 found for the boron atom, in line to their electronegativity difference (see Figure 2), as observed for U@C_27_B.^24^

**Figure 2:**
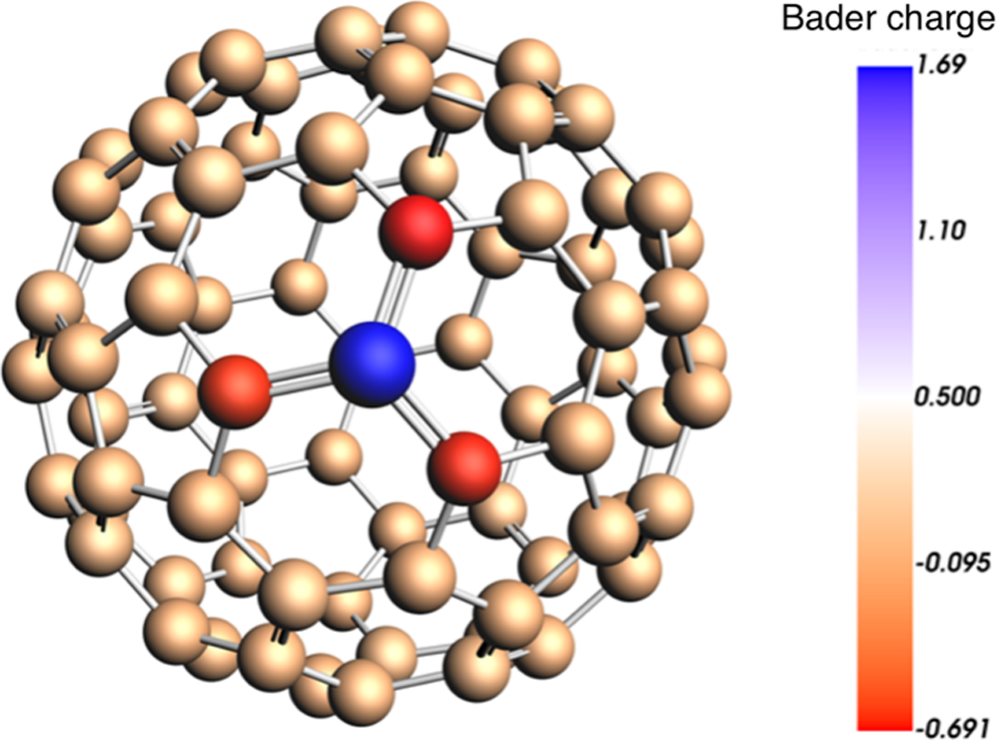
Bader charges for the C_79_B^6–^ 666 fullerene isomer.

### Sc_3_N@C_79_B: Cluster Orientation and Electronic Structure

It is well-known that in Sc_3_N@*I_h_* (7)-C_80_ there is free rotation of the nitride cluster inside the cage.^27,53,54^ But, is this high degree of cluster rotation kept in the C_79_B cage? To answer this question, we have done geometry optimizations starting from different initial structures, where we have placed the cluster in different planes and angles to cover most of the internal surface of the fullerene. We have done this analysis for the two substitution patterns, 666 and 665, since their energy difference as hexa-anions is rather small. The most favored orientations are those in which one of the Sc atoms is pointing to the boron (Figures 3 and S5) since the metal prefers to be close to the area where the largest negative charge is concentrated. As for the hexa-anions, the isomer with the lowest energy is the one with the boron atom in a 666 position, in line with previous work.^50^ We identified two nearly degenerate structures, isomers 666 iso-1 and 666 iso-3 (Figure S5), separated by just 0.4 kcal·mol^–1^. The only difference between them lies in the positioning of the two scandium atoms that do not point toward the boron. As a result, rotation around the B–Sc–N axis is allowed. Other orientations are somewhat higher in energy. Conversely, the lowest-energy 665 isomer lies just 2.2 kcal·mol^–1^ above the lowest-energy 666 isomer, slightly increasing the energy gap between the 666 and 665 isomers by 0.7 kcal·mol^–1^ compared to the empty hexa-anions. For isomer 666 iso-1, the Sc–B distance is 2.375 Å, and the Sc–C distances for the three C atoms bonded to B are 2.277, 2.489, and 2.601 Å. These three rather different values reflect the fact that the Sc atom is actually pointing to the middle of one of the three B–C bonds.

**Figure 3:**
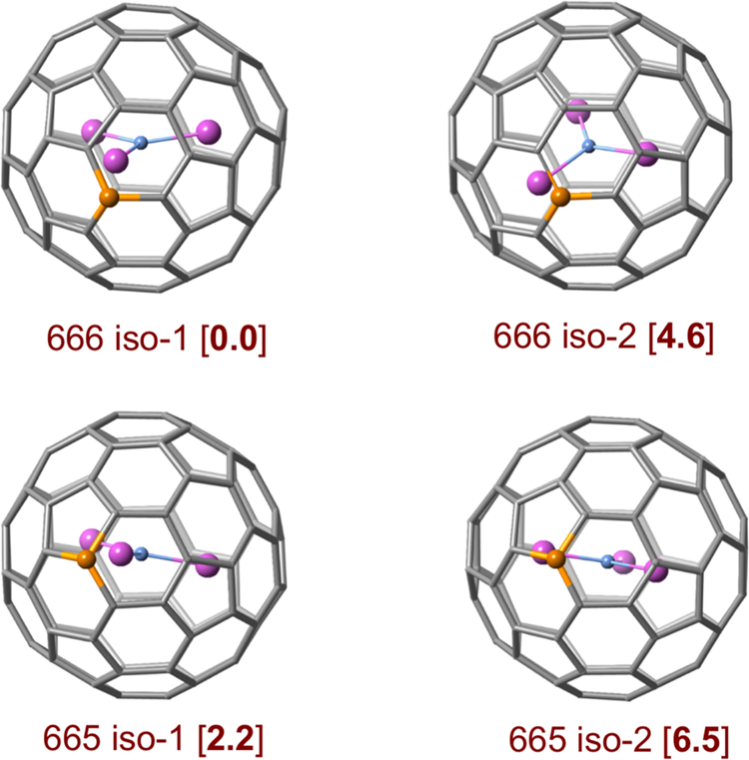
Representation of some of the computed isomers for 666 and 665 B-doped Sc_3_N@C_79_B clusterfullerenes with their relative energies indicated in square brackets (in kcal·mol^–1^). See Figure S5 for more details.

To further analyze the internal cluster rotation, we carried out CPMD simulations for both the boron-doped and all-carbon structures (see Computational Details Section) to compare the behavior of the cluster within each of the two cages. As initial geometry for Sc_3_N@C_79_B we chose the structure with the lowest energy from all those analyzed, that is, 666 iso-1. The simulations were carried out at 298 K during 25 ps. Figure 4 shows a different behavior of the cluster inside the two structures. In the boron-doped clusterfullerene, one of the scandium atoms remains essentially fixed, pointing toward the boron atom. Meanwhile, the rest of the cluster exhibits greater mobility, constrained only by the fixed position of one of the three scandium atoms. This is in line with the results found by static DFT calculations, which indicate that one of the metals remains near the boron while the other two can move around the B–Sc–N axis. In the all-carbon cage, the cluster moves with significantly greater freedom inside the fullerene cavity, as observed from NMR spectrum and previous calculations.^27,53,54^

**Figure 4:**
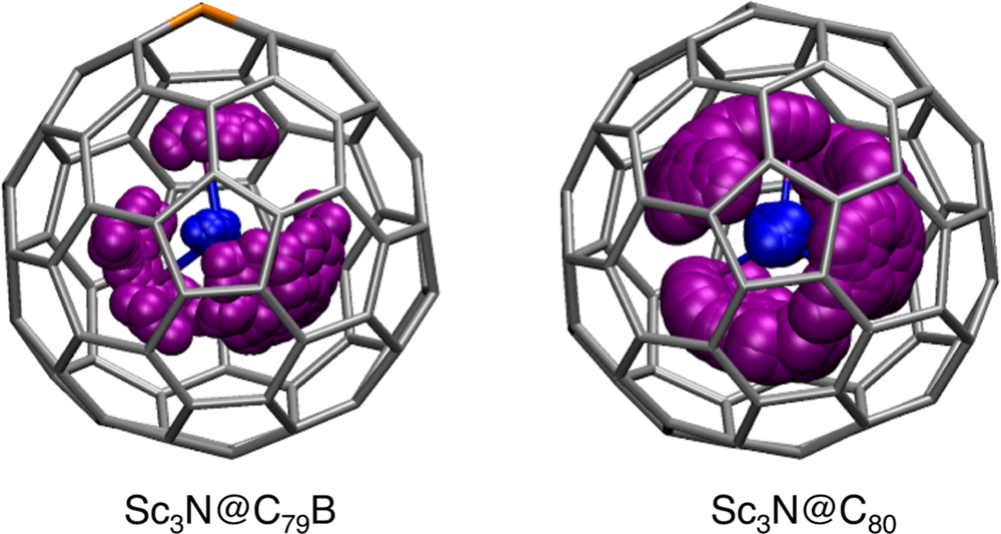
Motion of the Sc_3_N cluster inside the C_80_ and C_79_B (666) fullerene cages along the 25 ps trajectory of the Car–Parrinello MD simulations.

Finally, we have analyzed the electronic structure of this boron-doped nitride clusterfullerene and compared it to the all-carbon cage. Since Sc_3_N@C_79_B has one electron less than Sc_3_N@C_80_, it presents an open-shell electronic structure in which the single-occupied molecular orbital (SOMO) has a very similar energy to the HOMO of the Sc_3_N@C_80_ (Figure 5). Consequently, the boron-containing cage shows a very deep lowest-unoccupied molecular orbital (LUMO)-β (−5.45 eV compared to −5.56 eV of the SOMO) becoming a very good electron acceptor. Indeed, the computed electron affinity of Sc_3_N@C_79_B is 4.05 eV, compared to 2.67 eV for Sc_3_N@C_80_. In general, B-doped fullerene cages are good electron acceptors as previously found for C_59_B.^16^

**Figure 5:**
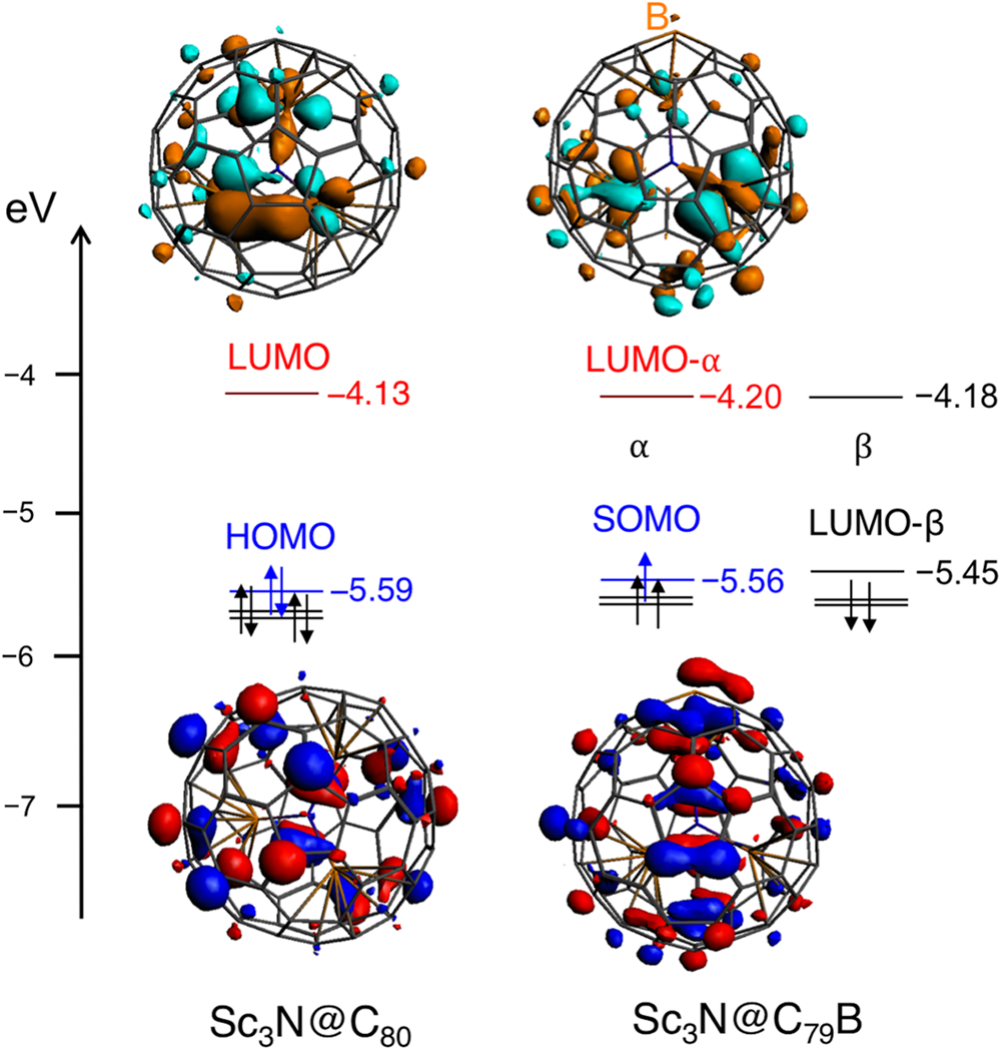
Frontier molecular orbitals for all-carbon Sc_3_N@C_80_ and the boron-doped Sc_3_N@C_79_B fullerene cage. The LUMOs surfaces are represented in light blue and orange and the HOMOs ones in dark blue and red.

### Higher Levels of B-Doping: Sc_3_N@C_78_B_2_ and Sc_3_N@C_77_B_3_

After analyzing the Sc_3_N@C_79_B clusterfullerene, we have considered the second replacement of a carbon with a boron, i.e., Sc_3_N@C_78_B_2_. First, we studied the hexa-anion cage. Once the first boron is placed in the carbon framework, the symmetry of the system is reduced and there is a larger number of possibilities for the second replacement. We fixed the first boron in the most stable position found, 666 position (#57), and analyzed the second substitution within one section of the cage, which covers some of the most representative nonequivalent C atoms (Figure S6). The results are summarized in Table S1. The lowest-energy C_78_B_2_^6–^ isomer, 666-iso-1, shows the two boron atoms at 666 sites in the same hexagon, in the *para*-relative position. We find other isomers at rather low energies (below 5 kcal mol^–1^) with the second B atom in either the 666 or 665 positions. When fixing the first B atom at a 665 position, the lowest-energy C_78_B_2_^6–^ isomer is found at 3.2 kcal mol^–1^ and the other computed isomers are at energies higher than 5 kcal mol^–1^ (Table S1 and Figure S6). From these results, it is remarkable that those isomers with contiguous B–B bonds show energies around 20 kcal·mol^–1^ above the lowest-energy structure. Bader charges for the lowest-energy hexa-anion, 666-iso-1 (Figure S6b) show that, in line with the results of the monodoped systems, the carbon atoms bonded to the two boron atoms are highly charged, with values ranging from −0.66 to −0.70.

Upon incorporation of the Sc_3_N cluster inside the fullerene, the situation changes slightly. The lowest-energy isomer, Sc_3_N@C_78_B_2_-iso-1, shows two of the Sc atoms directly pointing toward the B atoms located at 666 sites within the fullerene framework. Two structures with the B atoms in relative *para* positions are observed at around 3 kcal·mol^–1^ (Table 1 and Figure 6). The main difference between them is that in the case of Sc_3_N@C_78_B_2_-iso-2_1_, one Sc atom points to the center of the hexagon containing the two boron atoms, while in iso-2_2_, two Sc atoms point toward the B atoms, albeit less effectively, due to the constraints imposed by the cluster structure (Figure S7). Other isomers with one boron at a 666 site and the other one at a 665 site span an energy range between 5 and 12 kcal·mol^–1^. The relative energies for cages with two neighboring boron atoms exceed 20 kcal·mol^–1^, making it highly unlikely that this arrangement corresponds to the position of the boron atoms in the clusterfullerenes formed during the experiments.

**Figure 6:**
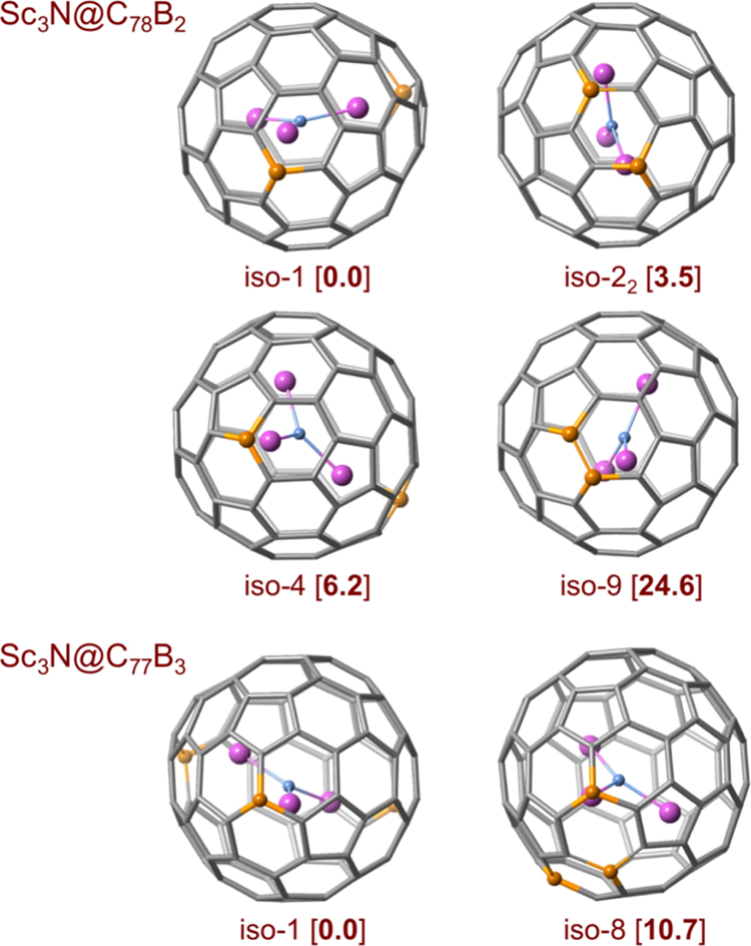
Representation of some Sc_3_N@C_78_B_2_ and Sc_3_N@C_77_B_3_ structures with their relative energies (kcal·mol^–1^) indicated. See SI for more details.

**Table 1:** Relative Energies for Di-Doped Sc_3_N@C_78_B_2_ Isomers^a^

isomer	B sites	position	Rel. energy
Sc_3_N@C_78_B_2_-iso-1	666–666		0.0
Sc_3_N@C_78_B_2_-iso-2_1_	666–666	*para*	3.1
Sc_3_N@C_78_B_2_-iso-3	666–665		4.5
Sc_3_N@C_78_B_2_-iso-4	666–665		6.2
Sc_3_N@C_78_B_2_-iso-5	666–665	*meta*	7.3
Sc_3_N@C_78_B_2_-iso-6	666–665	*meta*	11.7
Sc_3_N@C_78_B_2_-iso-7	665–665	*para*	15.3
Sc_3_N@C_78_B_2_-iso-8	666–665	*ortho*	23.3
Sc_3_N@C_78_B_2_-iso-9	666–665	*ortho*	24.6
Sc_3_N@C_78_B_2_-iso-10	665–665	*ortho*	27.7

a For a more detailed description of the isomers, see Table S2. Relative energies are in kcal mol^–1^.

To complete the analysis, we also made a preliminary study of the substitution of a third carbon in the clusterfullerene cage, although the peak corresponding to this fullerene had a very low abundance in the experiments. To simplify the problem, we have only considered the third replacement on the lowest-energy didoped system Sc_3_N@C_78_B_2_-iso-1 (Figure S8). The lowest-energy isomer found is one in which the third substitution also occurs on a 666 site (no. 30 in Table S3), Sc_3_N@C_77_B_3_-iso-1, in such a way that the three scandium atoms can target each of the boron atoms of the cage (Figure 6). Other positions close to and further from #30 were also analyzed. In general, (i) tridoped cages in which the B atoms can be stabilized by interaction with the three Sc atoms show rather low energies; (ii) isomers with three B atoms positioned at 666 sites show lower relative energies than those in which two B are at 666 sites and the third B is at a 665 site (Table S3).

### Synthesis of Boron-Doped Monometallofullerenes

Figures 7 and S10–S13 show fullerene synthesis products by laser evaporation of composite rods made by graphite, boron, and different metals (oxides) representative of Groups 2, 3, 4, lanthanides, and actinides: Ca, Sr, Sc, Y, La, Pr, Eu, Ho, Ti and Th. All experiments are performed under similar formation conditions that result in a complex mixture of metallofullerenes and empty cages formed through a bottom-up mechanism, as done in previous works.^55^ B-doped cages that encapsulate a metal atom range from Ti@C_27_B to Sr@C_79_B. Interestingly, the relative abundances of the B-doped fullerenes formed show an analogous behavior as the all-carbon cages, with peaks shifted to smaller cages when the charge transfer is increased.^55^ In contrast to all-carbon cages, however, the peak distributions in the spectra are shifted in general to larger cages (Figures S10–S20). For instance, for Y@C_2*n*_, the highest peaks appear at C_44_ and C_50_ with a small peak at C_60_. For Y@C_2*n*–1_B, the peak C_43_B is importantly decreased whereas the peak for C_59_B is increased. Something similar occurs for Th@C_2*n*_ and, in general, for the rest of the metals.

**Figure 7:**
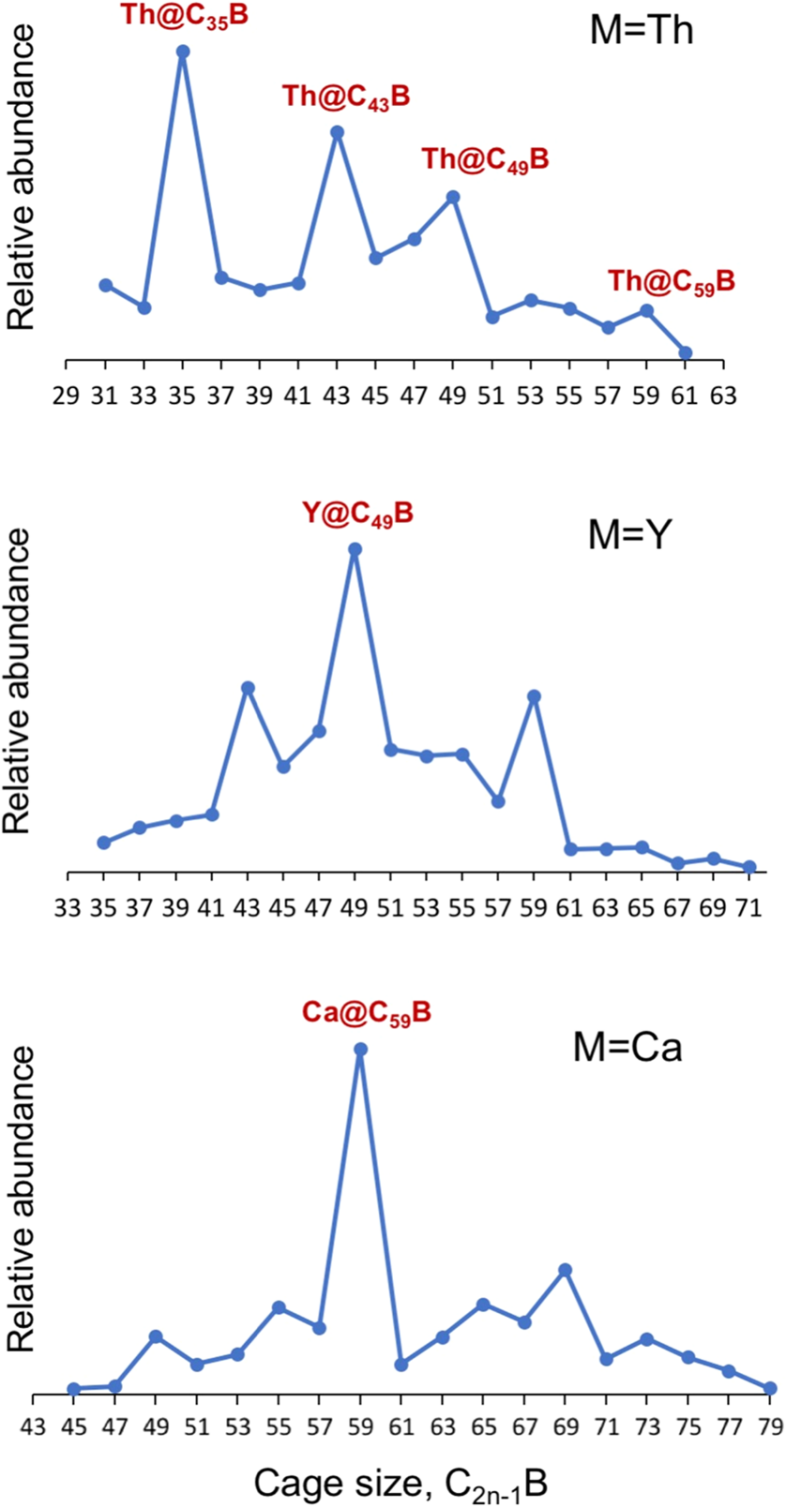
Relative abundances for different boron-doped monometallofullerene families M@C_2*n*–1_B (M = Ca, Y, Th) obtained by FT-ICR mass spectrometry. See Figures S10 and S13 for other metals.

To understand this behavior and to analyze the electronic structure of these B-doped fullerenes, we have studied in detail by means of DFT several M@C_59_B, M@C_49_B and some smaller systems.

### M@C_59_B Boron-Doped Fullerenes

We analyzed the structure and the formal charge transfer for several M@C_59_B where M = Rb, Sr, Y, Ti, and Th. Since for C_60_ (*I*_*h*_) all 60 carbon atoms are symmetry equivalent, there is only one possible C_59_B heterocage. As shown in Table 2, the encapsulation energy for the group of metal atoms considered increases when there is an increase in the formal charge transfer from the metal to the cage. The system with the lowest encapsulation energy is Rb@C_59_B (3.02 eV), which has a charge transfer of +1, while the cage with the highest encapsulation energy (7.89 eV) shows the highest charge transfer (+4). It is also remarkable that the highly charged metals point to the boron atom, as found for the nitride clusterfullerene (Figure 8). For M = Rb, the metal is placed almost at the center of the cage, with a Rb–B distance of 3.38 Å. In contrast, for Y and Ti, this distance decreases to 2.42 Å. For Th@C_59_B, the distance increases to 2.52 Å due to the larger size of Th.

**Figure 8:**
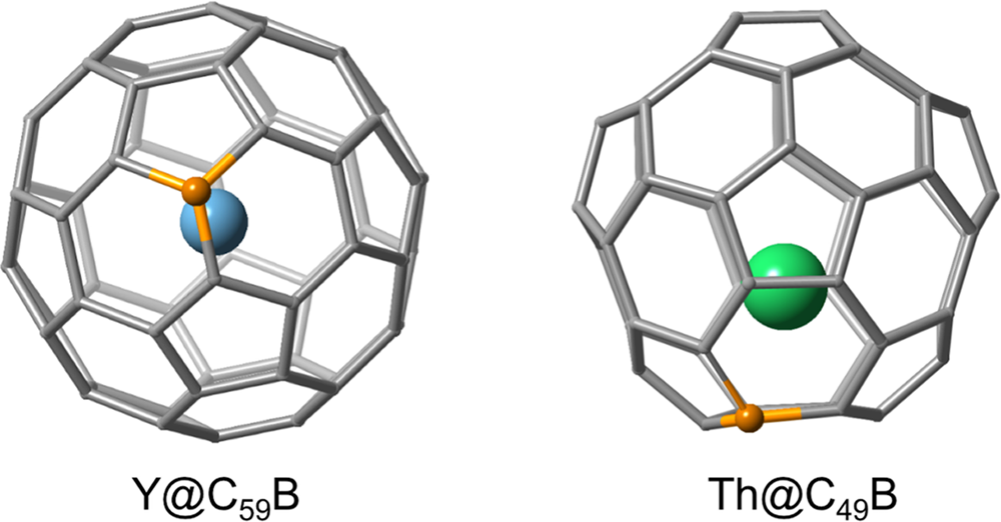
Representation of the optimized geometries of Y@C_59_B and Th@C_49_B. The C_59_B heterocage is based on *I_h_*-C_60_ and the C_49_B one on *D*_5*h*_-C_50_.

**Table 2:** Encapsulation Energies, Formal Charge Transfers and Metal–Boron and Metal–Cage Distances for M@C_59_B (M = Rb, Sr, Y, Ti and Th)^a^

M@C_59_B	Rb	Sr	Y	Ti	Th
EE	3.02	3.44	5.22	5.29	7.89
CT	1	2	3	3^c^	4
M–B	3.383	2.789	2.415	2.120	2.519
M–C^b^	3.32	2.83	2.45	2.17	2.54

a Encapsulation energies (EE) in eV; formal charge transfer (CT) in electrons; and distances in Å.

b Average M–C distance with nearest C atoms.

c Mulliken spin population on Ti is 0.6e.

Besides, we have explored the electronic structure of the M@C_59_B family and compared it to empty C_59_B heterocage and all-carbon *I_h_*-C_60_ (Figure 9). As noted, the radical C_59_B cage is a good electron acceptor since its LUMO is at a relatively quite low energy (−5.46 eV). When Rb is encapsulated inside the heterocage, the closed-shell electronic structure is re-established since now the heterocage (Rb^+^@C_59_B^–^) is isoelectronic to C_60_ fullerene. The HOMO of Rb@C_59_B is somewhat higher in energy than that in *I_h_*-C_60_ due to the symmetry breaking of the cage and the participation of boron, which is slightly less electronegative than carbon. Regarding the charges, we have confirmed that the three C atoms directly bonded to B show the highest negative charges in the cage. Sr@C_59_B, with a formal transfer of +2, has an electronic structure that is analogous to that of Rb@C_60_ with a charge transfer of +1. These systems, with an unpaired electron delocalized in the cage, show a low oxidation potential (see the SOMO energy of Sr@C_59_B in Figure 9). After this analysis, the results obtained in the mass spectrometry experiments could be better understood. The shift in abundances for B-doped monometallofullerenes toward larger masses, compared to those of all-carbon cages, is likely due to the lack of one electron in C_2*n*–1_B relative to C_2*n*_ cages. One of electrons that are transferred from the metal to the heterocage replaces this missing electron. This causes boron-doped monometallofullerenes with a charge transfer of +1 to behave as all-carbon empty fullerenes. When the charge transfer is +2, they behave like all-carbon systems with a charge transfer of +1 and so on.

**Figure 9:**
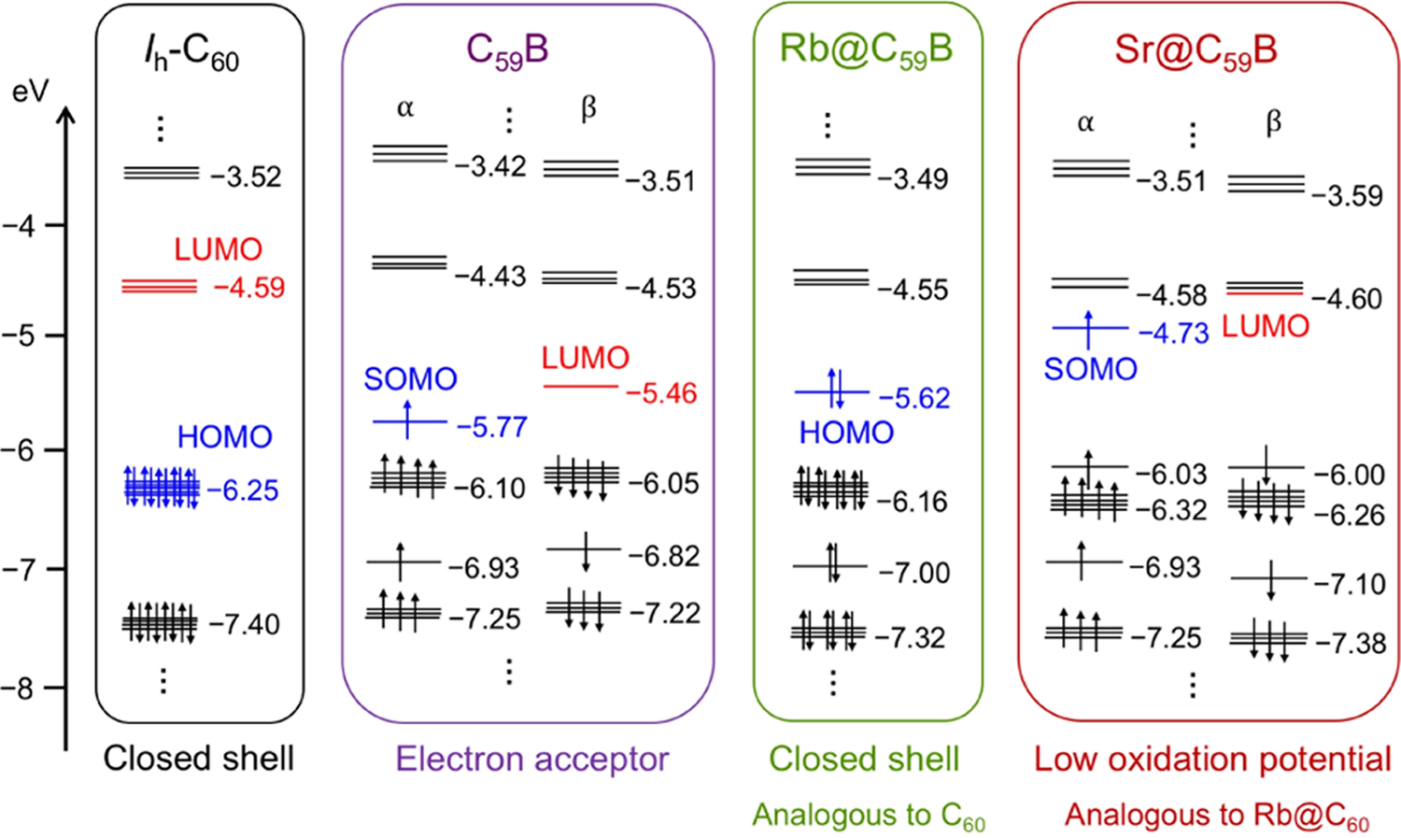
Representation of the frontier molecular orbitals energy diagram for C_60_, C_59_B, Rb@C_59_B, and Sr@C_59_B.

### M@C_49_B Boron-Doped Fullerenes

To corroborate the previous hypothesis, we did the same electronic and structural analysis for the M@C_49_B family where M = Y and Th. Now, for C_49_B we have chosen cage *D*_5*h*_-C_50_ because it is a well-studied isomer from C_50_ family due to its stability and properties.^55,56^ Different positions for the B atom were considered. The results obtained for the lowest-energy structures are summarized in Table 3.

**Table 3:** Encapsulation Energies, Formal Charge Transfers, and Metal–Boron and Metal–Cage Distances for M@C_49_B (M = Y and Th)^a^

M@C_49_B	Y	Th
EE	5.57	8.07
CT	3	4
M–B	2.458	2.616
M–C^b^	2.49	2.59

a Encapsulation energies (EE) in eV; Formal charge transfer (CT) in electrons; and distances in Å.

b Average M–C distance with nearest C atoms.

A similar pattern is observed for these two monometallofullerenes when compared to M@C_59_B. Encapsulation energies increase when formal charge transfer is increased. They are rather similar (slightly larger) to those found for the corresponding M@C_59_B systems. Besides, the metal formally transfers 3 (Y) or 4 (Th) electrons to the cage, and therefore, the metal atom is close to the boron atom (2.46 and 2.63 Å for Y and Th, respectively, Figure 8). The M–C distances are rather similar.

We have analyzed the electronic structure of these two B-doped fullerenes and compared them with the all-carbon cage Ca@C_50_, where two electrons are transferred from the calcium atom to the fullerene (Figure 10). Y@C_49_B shows an electronic structure almost identical to that of Ca@C_50_, with very similar orbital energies. Therefore, yttrium metal, which transfers three electrons to the boron-doped cage, behaves like calcium in a boron-free fullerene. This is consistent with the observed shift of abundances in the cases of boron-doped monometallofullerenes compared to all-carbon cages. Finally, we note that radical Th@C_49_B shows a low oxidation potential, with ε_SOMO_ = −4.56 eV, which is even higher than that of Sr@C_59_B (ε_SOMO_ = −4.73 eV).

**Figure 10:**
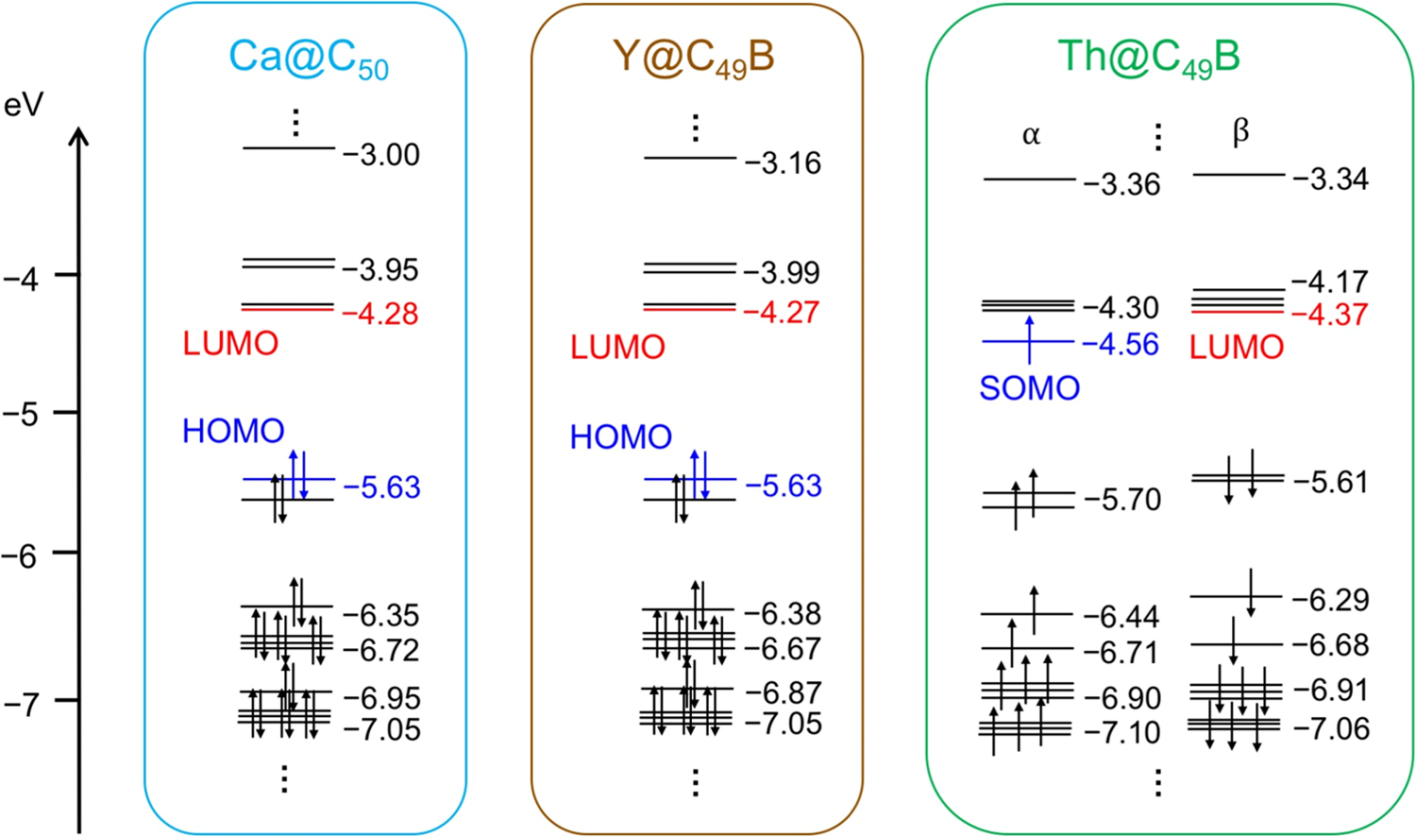
Representation of the frontier molecular orbitals energy diagram for Ca@C_50_, M@C_49_B where M = Y and Th.

### Th@C_43_B and Th@C_35_B Boron-Doped Fullerenes

We have analyzed the two most intense peaks that appear in the FT-ICR mass spectrum for small cage sizes in the Th@C_2*n*–1_B family, Th@C_43_B and Th@C_35_B (see Figure 7). For Th@C_43_B, we considered the cages with the lowest energies in Ti@C_44_, which have the same formal electron transfer. These isomers are *D*_2_-C_44_(75) and *D*_2_-C_44_(89).^56^ Same procedure was used for Th@C_35_B, which leads to isomers *D*_2*d*_-C_36_(14) and *D*_6*h*_-C_36_(15). Different positions for the B atom were considered. The results obtained for the lowest-energy structures are summarized in Table 4. For all of the systems, the unpaired electron is delocalized on the heterocage. The encapsulation energies increase by more than 1 eV with respect to C_49_B cages. Interestingly, the Th–B and Th–C distances increase, especially for Th@C_35_B. This result, which seems counterintuitive at first, arises from the displacement of the Th ion from near the wall to the center of the cage as the cage size decreases (Figure 9). As a result, Th can interact with the maximum number of C atoms, as observed in U@C_27_B.^24^

**Table 4:** Encapsulation Energies, Formal Charge Transfers, and Metal–Boron and Metal–Cage Distances for Th@C_43_B and Th@C_35_B^a^

Th@C_2*n*–1_B	C_43_B(75)	C_35_B(14)	C_35_B(15)
EE	9.99	9.38	9.38
CT	4	4	4
Th–B	2.671	2.791	2.729
Th–C^b^	2.63	2.67	2.64

a Encapsulation energies (EE) in eV; Formal charge transfer (CT) in electrons; and distances in Å.

b Average Th–C distance with nearest C atoms.

### Ti@C_43_B, Ti@C_29_B, and Ti@C_27_B B-Doped Fullerenes

Finally, we have considered several intense peaks in the FT-ICR mass spectrum for the Ti@C_2*n*–1_B family, that is, Ti@C_43_B and Ti@C_29_B, as well as the smallest observed heterocage Ti@C_27_B (see Figure S10). As for Th@C_43_B, we have chosen cages *D*_2_-C_44_(75) and *D*_2_-C_44_(89). One isomer based on C_43_B­(75) with B on the 566 position shows the lowest energy. For C_30_, we have studied the three possible isomers.^57^ B-doped cage based on *C*_2*v*_-C_30_(3) presents the lowest energy, 15 kcal mol^–1^ lower than that of B-doped *C*_2*v*_-C_30_(2) systems. The lowest-energy Ti@C_29_B that we have found shows the B atom placed at a 566 position (Figure 11). The Ti atom interacts with fused pentagons at around 2.11 Å and at a distance from the B atom, 2.642 Å, which is somewhat larger than in other systems. Other low-energy isomers of the C_29_B­(3) cage show Ti–B distances around 2.1–2.2 Å. The Ti@C_27_B isomers are based on the *T*_d_-C_28_(2) cage, as for U@C_27_B. Lower energy is found when boron is at the 556 position, which is 6.5 kcal mol^–1^ lower than when placed at the 555 position. Consistent with Ti@C_28_ and in contrast to U@C_27_B, the Ti atom is off-centered, positioned 2.149 Å from the B atom and 2.13 Å on average from the nearest cage atoms. In all these small B-doped monotitanofullerenes, the formal charge transfer is four. The encapsulation energies are significantly larger than those in Ti@C_59_B, in line with the increased charge transfer (4 vs 3) and smaller cage sizes. The quite small value of 6.82 eV found for Ti@C_29_B­(3) is due to the higher relative stability of the host heterocage, which is between 0.5 and 1 eV lower in energy than other isomers (Figure 11 and Table 5).

**Figure 11:**
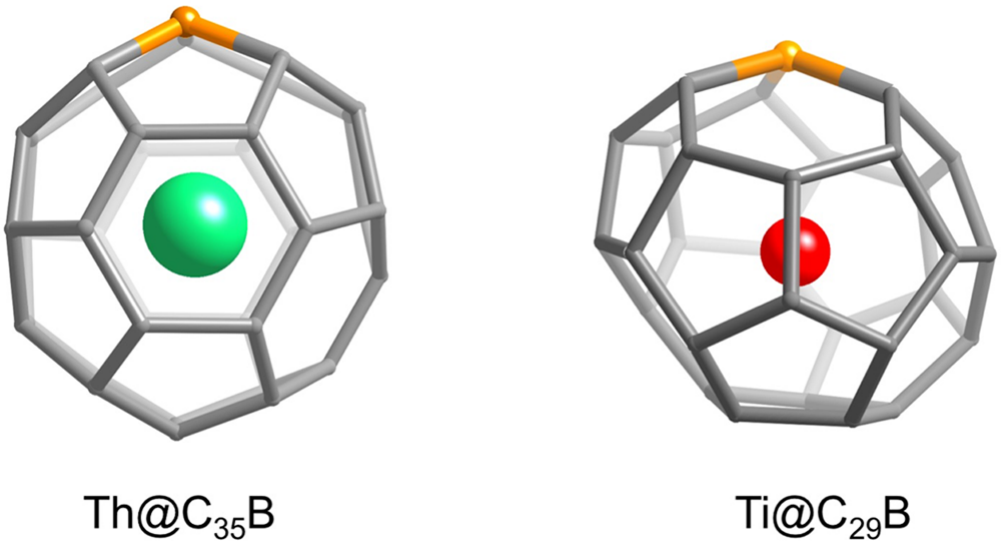
Representation of the optimized geometries of Th@C_35_B and Ti@C_29_B. The C_35_B heterocage is based on *D*_2_-C_36_(14) and the C_29_B one on *C*_2*v*_-C_30_(3).

**Table 5:** Encapsulation Energies, Formal Charge Transfers, and Metal–Boron and Metal–Cage Distances for Ti@C_43_B, Ti@C_29_B, and Ti@C_27_B^a^

Ti@C_2n‑1_B	C_43_B(75)	C_29_B(3)	C_27_B(2)
EE	7.49	6.82	8.79
CT	4	4	4
Ti–B	2.209	2.642	2.149
Ti–C^b^	2.13	2.11	2.13

a Encapsulation energies (EE) in eV; formal charge transfer (CT) in electrons; and distances in Å.

b Average Ti–C distance with nearest C atoms.

## Conclusions

After doping the iconic C_60_ fullerene, C_59_B, and the smallest endohedral metallofullerene, U@C_27_B, boron-doped nitride clusterfullerenes Sc_3_N@C_80–*x*_B_*x*_ and Sc_3_N@C_80_B_*x*_ (*x* = 1–3), along with a series of monometallofullerenes M@C_2*n*–1_B (M=Ca, Sr, Sc, Y, La, Pr, Eu, Ho, Ti, Th) are produced using laser vaporization techniques and detected by high-resolution FT-ICR mass spectrometry through two different routes. Clusterfullerenes were obtained from pre-existing Sc_3_N@C_80_ exposed to B-containing vapor through atom exchange and monometallofullerenes from starting carbon/boron materials through the bottom-up formation mechanism. We have found that the carbon atoms contiguous to boron are highly charged, as seen from Bader charge analysis. In both boron-doped nitride clusterfullerenes and monometallic cages, the metal atoms are located near the boron atom, except in the case of Rb@C_59_B, where the Rb^+^ ion remains more centered within the cage. We have also observed that endohedral B-doped fullerenes, such as Sc_3_N@C_79_B, are good electron acceptor molecules. Additionally, boron–boron bonds in Sc_3_N@C_78_B_2_ are avoided due to the destabilizing effect these bonds have on the fullerene cage. Other endohedral B-doped fullerenes, such as Sr@C_59_B, which is isolectronic with Rb@C_60_, show low oxidation potentials. Finally, we have seen that the different electronic structures of the heterocages lead to altered formation distributions. This is likely because, in general, the electronic structures of M­(q + 1)@C_2*n*–1_B are analogous to those of M′(q)@C_2*n*_. Scaling-up the formation of these metalloheterofullerenes would be beneficial to exploring and confirm their interesting properties and potential applications, especially in comparison to their all-carbon counterparts.

## Experimental Section

### Synthesis and Isolation

#### Monometallic M@C_2*n*–1_B Systems

The starting materials, graphite (99.9999%), boron powder (96%), and metal oxides, are thoroughly mixed and then molded into a composite rod by compression. The B-containing M@C_2*n*–1_B are formed, with 10% B (atomic percent) and 1% metal (atomic percent), *in situ* by use of a pulsed supersonic cluster source by a single laser pulse of a Nd:YAG laser (532 nm, 5 mj/pulse) under a flow of helium.^55,58^ The gas-phase reaction products were analyzed by a custom-built 9.4 T FT-ICR mass spectrometer directly coupled to the cluster source and are conducted with positive ions.^52^ Ions produced by 10 individual vaporization events were accumulated and transferred by octopoles to an open cylindrical trap ICR cell. The ions are then accelerated to a detectable radius by a broadband frequency sweep excitation and detected as the differential current induced between two opposed electrodes of the ICR cell. The positively charged molecular ions are expected to be representative of the neutral abundance distribution generated by laser vaporization. However, we note that the corresponding neutrals may exhibit different stabilities. The boron-containing monometallic endofullerenes are formed from the boron- and metal-doped graphite starting material through the bottom-up formation mechanism.^52,55,16^

#### Trimetallic Nitride Sc_3_N@C_80–*x*_B*_x_* and Sc_3_N@C_80_B*_x_* Systems (*x* = 1–3)

Isomerically pure Sc_3_N@*I_h_*-C_80_ was uniformly applied to the surface of a target rod that contains a 1:1 atom ratio of C/B (from the usual graphite and B powder used) for plasma exposure studies by use of a Nd:YAG laser (532 nm, 15 mJ per pulse) cluster source. The gas-phase reaction products were analyzed by a custom-built 9.4 T FT-ICR mass spectrometer directly coupled to the cluster source and are conducted with positive ions (same settings as for previous case).^52^ The boron-containing clusterfullerenes grow from the pre-existing Sc_3_N@C_80_ cage through atom exchange and bottom-up boron incorporation events.^16^

## Computational Details

All calculations were carried out using density functional theory (DFT) with the ADF 2018 package^59,60^ using BP86 exchange-correlation functional.^61,62^ Slater triple-ζ polarization (TZP) basis sets were used to describe the valence electrons of Rb, Sr, Y, Ti, Th, Sc, C, N, and B.^63,64^ Frozen cores were described by means of single Slater functions, consisting of the 1s shell for C, N, and B; the 1s to 2p shells for Sc and Ti; the 1s to 3d shells for Rb, Sr, and Y; and the 1s to 5d shells for Th. Scalar relativistic corrections were included by means of the ZORA formalism. Dispersion corrections by Grimme were also included.^65^ Open-shell calculations were performed at an unrestricted level. Car–Parrinello molecular dynamics simulations were done using the CPMD code.^66^ The description of the electronic structure was based on the expansion of the valence electronic wave functions into a plane wave basis set, which was limited by an energy cutoff of 100 Ry. The interaction between the valence electrons and ionic cores was treated through the pseudopotential (PP) approximation. Martins–Troullier PP was used for C, N, and B and Goedecker-Teter–Hutter PP for Sc.^67–70^ The functional PBE was selected as the density functional,^71^ and dispersion corrections (Grimme) were considered. The Nosé–Hoover thermostat for the nuclear degrees of freedom was used to maintain the temperature as constant as possible (298 K). In all simulations, the wave function is converged at the beginning of the MD run. The simulations were carried out in a cubic cell with a side length of 20 Å and a time step of 0.144 fs.

## Supplementary Material



## Notes

Additional computational and experimental results about boron-doped scandium nitride clusterfullerenes; MS spectra and relative abundances for boron-doped monometallofullerenes M@C_2*n*–1_B and Cartesian coordinates for optimized structures (PDF)
