# Supplementary material for: Boron-Doped Endohedral Metallofullerenes: Synthesis and Computational Analysis of a Family of Heteroatom-Doped Molecular Carbons
Source: Inorg Chem. 2025 Jan 8;64(2):1208–17. doi: 10.1021/acs.inorgchem.4c05122 (PMC12435174; doi:10.1021/acs.inorgchem.4c05122)
Supplement: Supplementary file 1 [file ic4c05122_si_001.pdf]

Supporting Information

Boron-Doped Endohedral Metallofullerenes: Synthesis and Computational Analysis of a Family of Heteroatom-Doped Molecular Carbons

Antonio Moreno-Vicente,[a] Sven Schardt,[b,c] Paul W. Dunk,[d] Josep M. Poblet,[a] Antonio Rodríguez-Fortea,*,[a]

[a]Departament de Química Física i Inorgànica, Universitat Rovira i Virgili, c/Marcel·lí Domingo 1, 43007 Tarragona, Spain

[b]Department of Chemistry, Technische Universität Darmstadt, Alarich-Weiss-Straße 8, 64287 Darmstadt, Germany

[c]Karlsruhe Institute of Technology, Institute for Chemical Technology and Polymer Chemistry, Engesserstr. 20, 76131 Karlsruhe, Germany

[d]National High Magnetic Field Laboratory, Florida State University, 1800 East Paul Dirac Drive, Tallahassee, FL 32310, USA

Figure S1. FT-ICR mass spectrum of $Sc_3N@C_{80}$ and $Sc_3N@C_{80-x}B_x$ (x=1,2)....................2

Figure S2. Simulated mass spectrum for $Sc_3N@C_{80}B_x$ (x=1-3)..2

Figure S3. The two non-symmetric C atoms on I_h(7)-C_{80} cage...2

Figure S4. Spin density for the two different $C_{79}B^{6-}$& Bader charges for $C_{79}B$ $(666)^{6-}$.......3

Figure S5. Four most stable isomers for 666 and 665 of $Sc_3N@C_{79}B$ 3

Figure S6. Different di-doped systems considered & Bader charges for $C_{78}B_2^{6-}$................4

Table S1. Relative energies for computed $C_{78}B_2^{6-}$ systems..4

Figure S7. Different $Sc_3N@C_{78}B_2$ considered & Structures of some of them....................5

Table S2. Relative energies for computed $Sc_3N@C_{78}B_2$ systems 5

Figure S8. Different $Sc_3N@C_{77}B_3$ considered 6

Table S3. Relative energies for computed $Sc_3N@C_{77}B_3$ systems 6

Figure S9. Structures of computed $Sc_3N@C_{77}B_3$ systems 6

Figure S10-S13. Abundances for $M@C_{2n-1}B$ in FT-ICR mass spectrometry. 7-8

Figure S14-S20. Mass spectra of $M@C_{2n-1}B$ 9-15

xyz coordinates of optimized structures...16

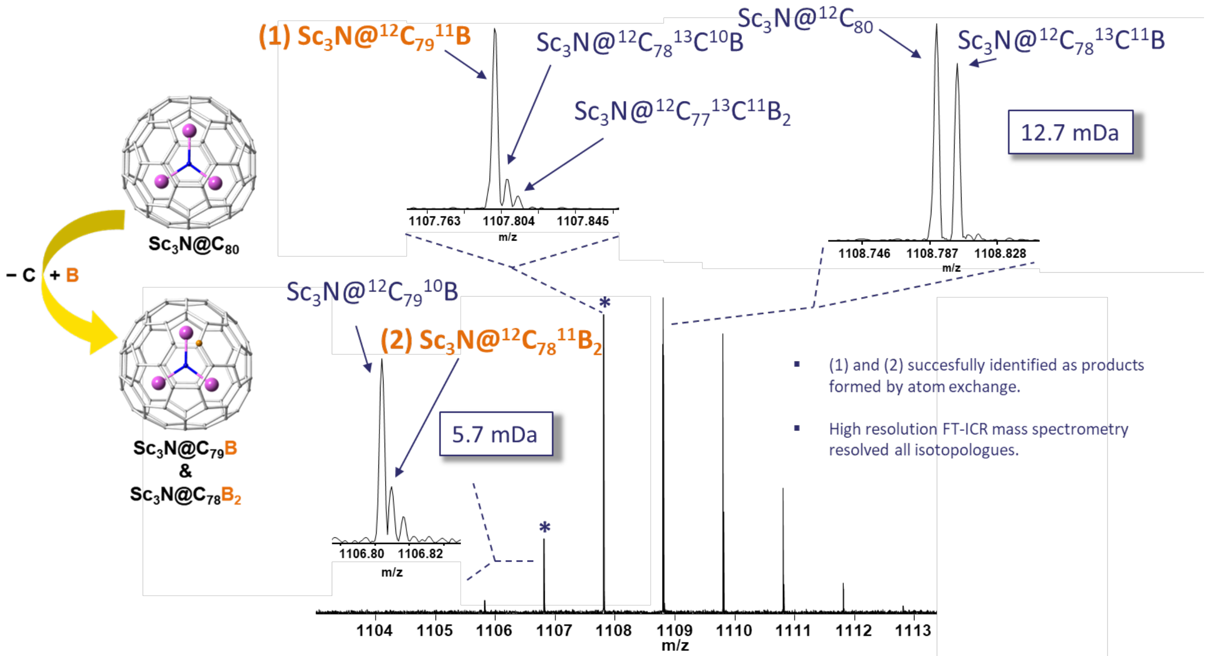

Figure S1. High-resolution FT-ICR mass spectrum of $Sc_3N@C_{80}$, $Sc_3N@C_{79}B$ and $Sc_3N@C_{78}B_2$ and their isotopologues.

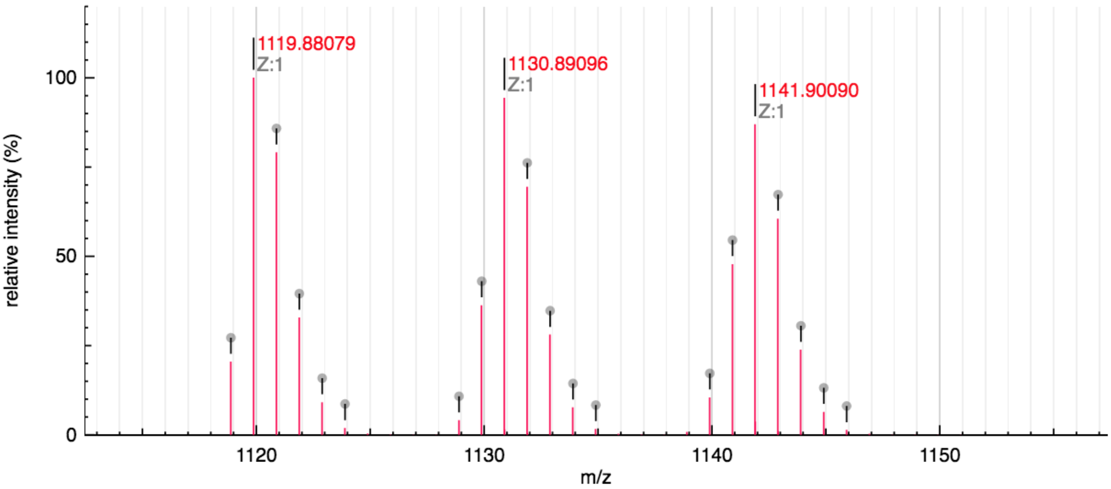

Figure S2. Simulated mass spectrum for $Sc_3N@C_{80}B$, $Sc_3N@C_{80}B_2$ and $Sc_3N@C_{80}B_3$.

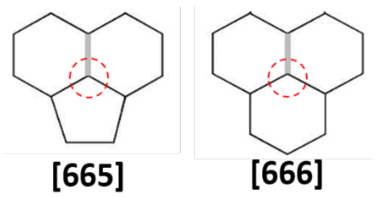

Figure S3. The only two non-symmetric C atoms on the $I_h(7)$-C_{80} fullerene cage.

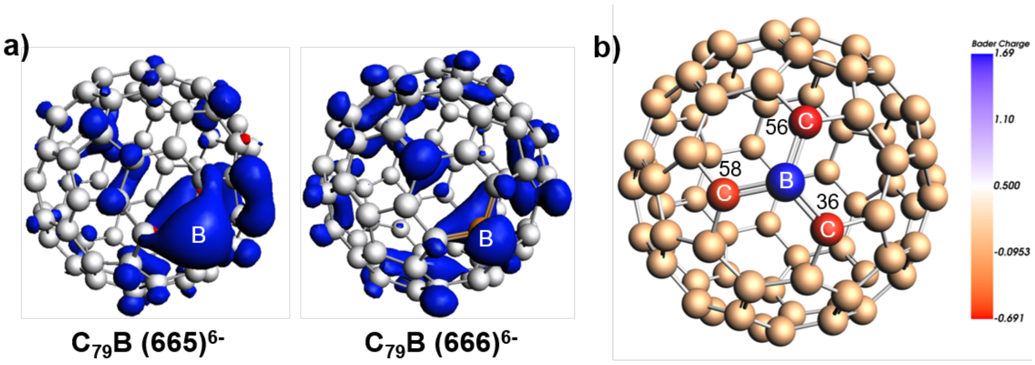

Figure S4. a) Representation of the spin density for the two different hexa-anion isomers; b) Representation of the Bader charges for the $C_{79}B$ (666)$^{6-}$ fullerene. Charges on B and three bonded C atoms are: 1.69 (B), -0.67 (C36), -0.69 (C56) and -0.65 (C58).

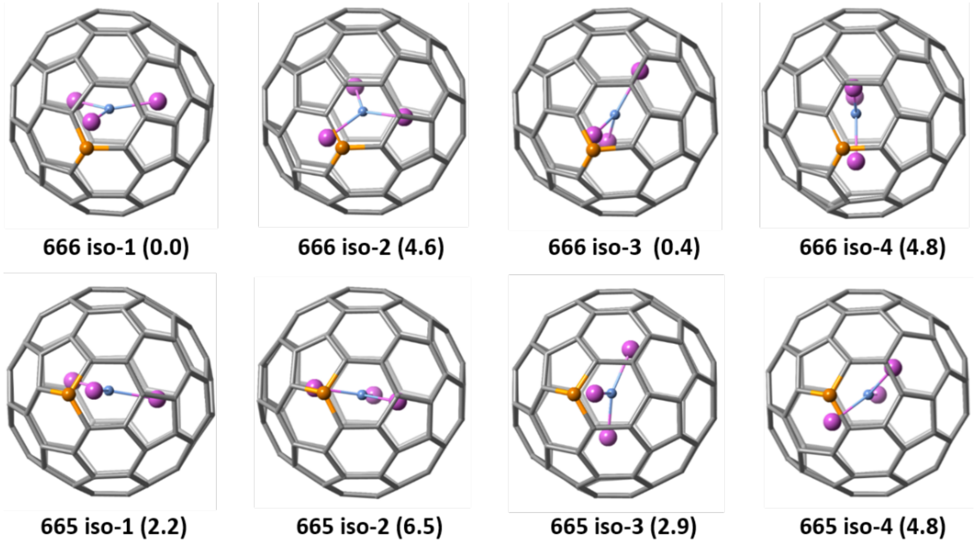

Figure S5. Representation of the four most stable computed isomers for 666 and 665 B-doped clusterfullerenes with their relative energy indicated in parenthesis (in kcal·mol^{-1}).

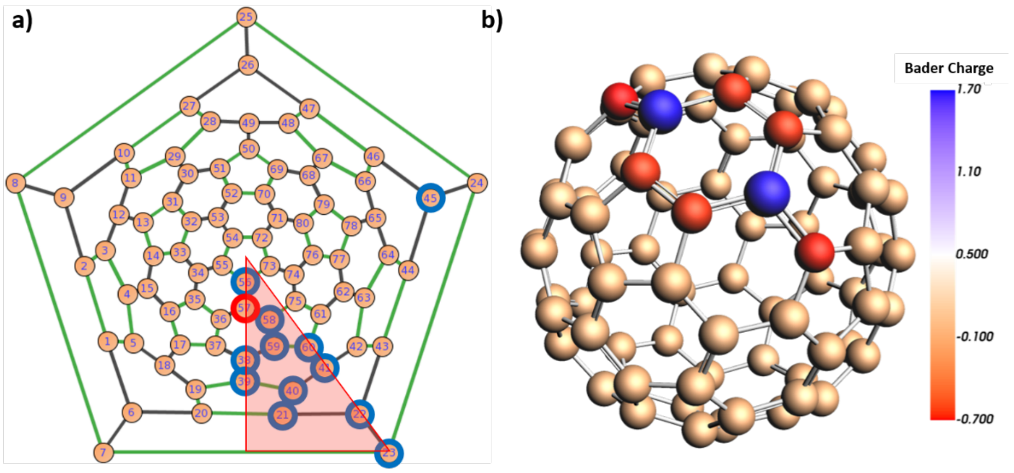

Figure S6. a) Schlegel diagram of the $I_h(7)$-C_{80} fullerene cage. The red triangle represents the area selected for the addition of the second boron atom. All the pentagons are highlighted in green. The red circle represents the position of the fixed boron atom and the blue circles the positions analysed for the second substitution. b) Representation of the Bader charge analysis of the most stable $C_{78}B_2^{6-}$ isomer.

Table S1. Relative energies (RE) for the substitution of the second carbon atom by boron in fullerene $C_{79}B^{6-}$ to give $C_{78}B_2^{6-}$.

**Iso 666**[a]	**2nd B position**	**RE**[c]	**Iso 665**[b]	**2nd B position**	**RE**[c]
666-iso-1	38	0.0	665-iso-1	75	3.2
666-iso-2	45	2.7	665-iso-2	22	5.5
666-iso-3	21	4.3	665-iso-3	58	6.5
666-iso-4	40	4.8	665-iso-4	38	6.6
666-iso-5	23	5.6	665-iso-5	23	7.8
666-iso-6	59	5.9	665-iso-6	39	8.3
666-iso-7	22	6.4	665-iso-7	60	9.4
666-iso-8	39	6.7	665-iso-8	73	19.9
666-iso-9	41	7.9			
666-iso-10	60	8.1			
666-iso-11	58	21.5			
666-iso-12	56	21.6			

[a] 1st boron at 666 position #57. [b] 1st boron at 665 position #56. [c] Energies in $kcal \cdot mol^{-1}$.

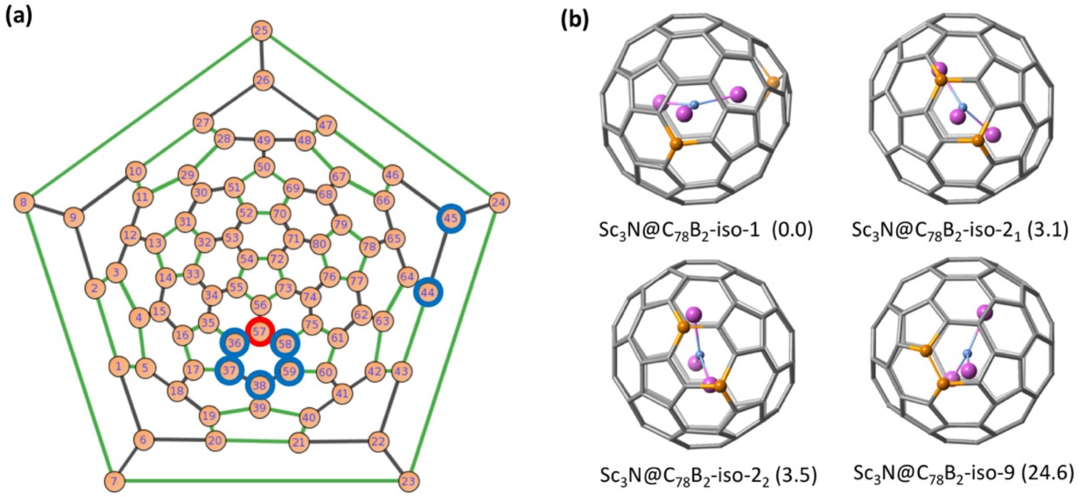

Figure S7. a) Schlegel diagram of the I_h(7)-C_{80} fullerene cage. All the pentagons are highlighted in green. The red circle represents the position of the fixed boron atom at a 666 site and the blue circles the positions analysed for the second boron. b) Representation of some $Sc_3N@C_{78}B_2$ structures with their relative energies (kcal·mol^{-1}) indicated.

Table S2. Relative energies (RE) for the substitution of the second carbon atom by boron in mono-doped $Sc_3N@C_{79}B$ to give di-doped $Sc_3N@C_{78}B_2$.

**Iso 666**[a]	**2nd B position**	**RE**[c]	**Iso 665**[b]	**2nd B position**	**RE**[c]
$Sc_3N@C_{78}B_2$-iso-1	45	0.0	$Sc_3N@C_{78}B_2$-iso-4	22	6.2
$Sc_3N@C_{78}B_2$-iso-2_1	38	3.1	$Sc_3N@C_{78}B_2$-iso-7	75	15.3
$Sc_3N@C_{78}B_2$-iso-2_2	38	3.5	$Sc_3N@C_{78}B_2$-iso-10	73	27.7
$Sc_3N@C_{78}B_2$-iso-3	44	4.5			
$Sc_3N@C_{78}B_2$-iso-2_3	38	4.7			
$Sc_3N@C_{78}B_2$-iso-5	37	7.3			
$Sc_3N@C_{78}B_2$-iso-6	59	11.7			
$Sc_3N@C_{78}B_2$-iso-8_1	58	23.3			
$Sc_3N@C_{78}B_2$-iso-9	36	24.6			
$Sc_3N@C_{78}B_2$-iso-8_2	58	26.1			

[a] 1st boron at 666 position #57. [b] 1st boron at 665 position #56. [c] Energies in kcal·mol^{-1}.

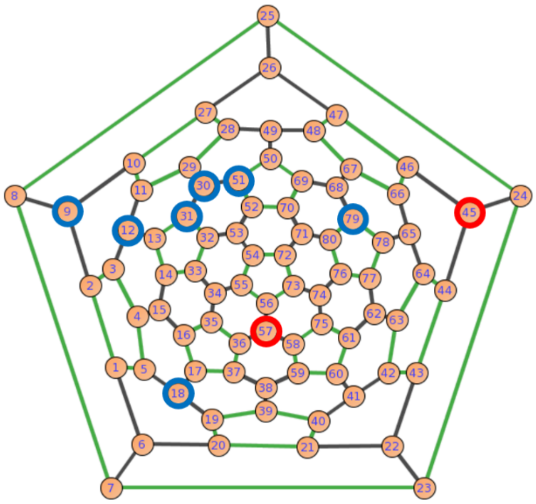

Figure S8. Schlegel diagram of the I_h(7)-C_{80} fullerene cage. All the pentagons are highlighted in green. The red circles represent the positions of the fixed boron atoms and the blue circles the positions analysed for the third boron in tri-doped $Sc_3N@C_{77}B_3$ isomers.

Table S3. Relative energies (RE) for the substitution of the third carbon atom by boron in di-doped $Sc_3N@C_{78}B_2$ to give tri-doped $Sc_3N@C_{77}B_3$.

**Iso 666**[a]	**3rd B position**	**RE**[b]	**Iso 666**[a]	**3rd B position**	**RE**[b]
$Sc_3N@C_{77}B_3$-iso-1	30	0.0	$Sc_3N@C_{77}B_3$-iso-5	51	7.5
Iso-2	12	0.8	Iso-6	18	7.8
Iso-3	9	1.7	Iso-7	79	7.9
Iso-4	31	3.7	Iso-8	[c]	10.7

[a] 1st and 2nd boron at 666 positions #57 and #45. [b] Energies in kcal·mol^{-1}. [c] Positions of the boron in iso-8 are #57, #38 and #18.

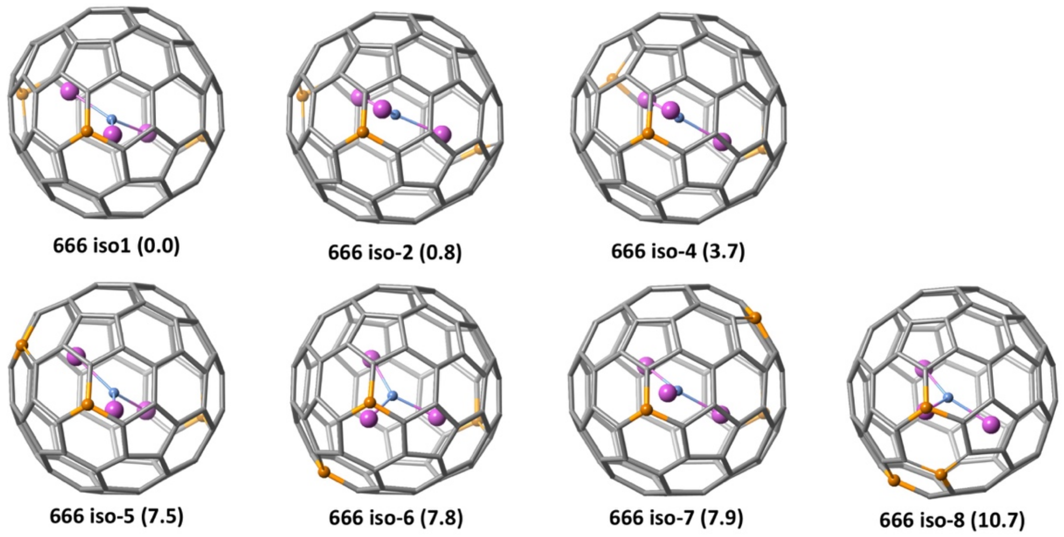

Figure S9. Representation of some of the computed isomers for 666 B-doped $Sc_3N@C_{77}B_3$ with their relative energy indicated (in kcal·mol^{-1}).

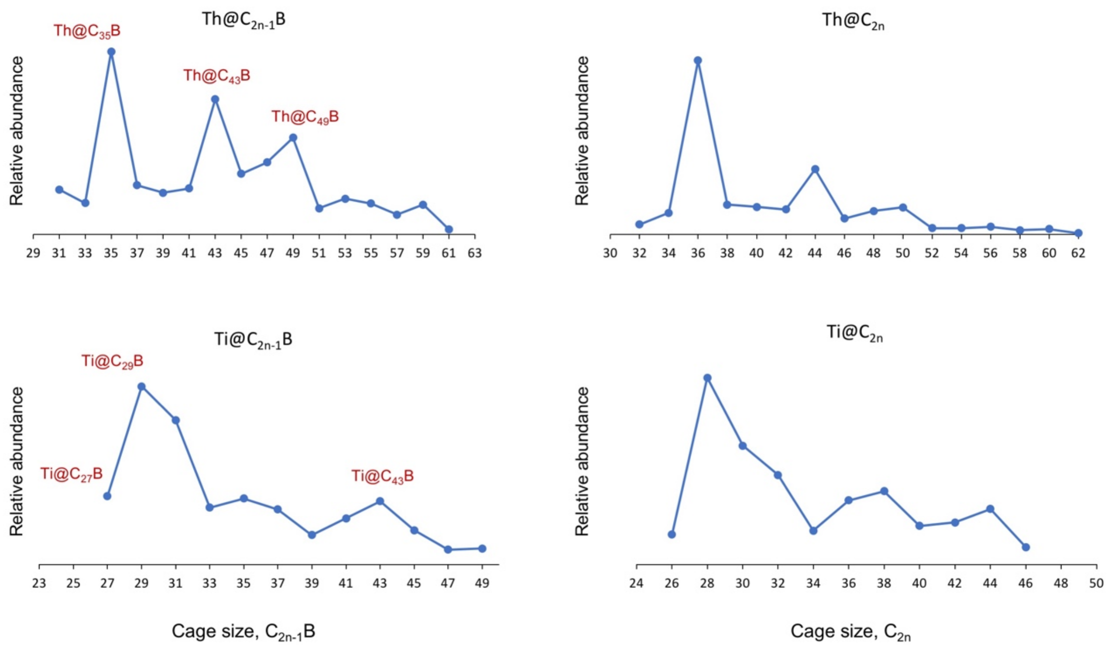

Figure S10. Relative abundances for boron-doped monometallofullerene families M@C_{2n-1}B (M = Th and Ti) obtained by FT-ICR mass spectrometry.

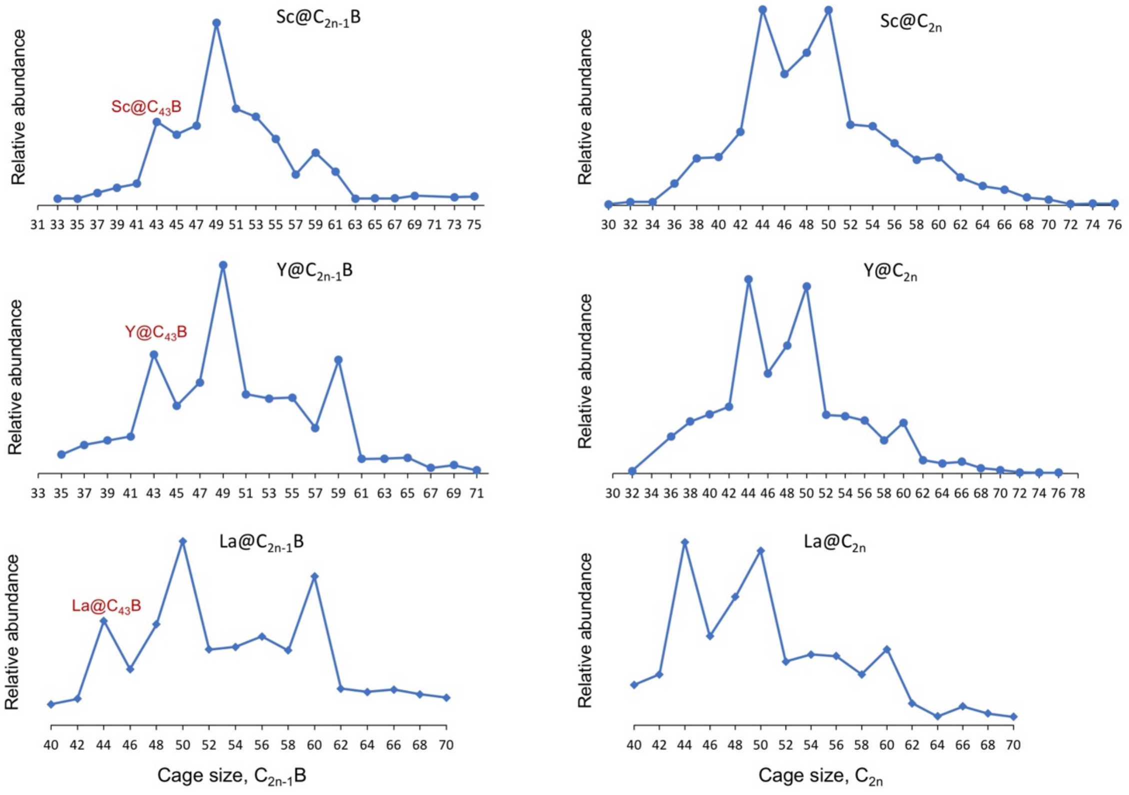

Figure S11. Relative abundances for boron-doped monometallofullerene families M@C_{2n-1}B (M = Sc, Y and La) obtained by FT-ICR mass spectrometry.

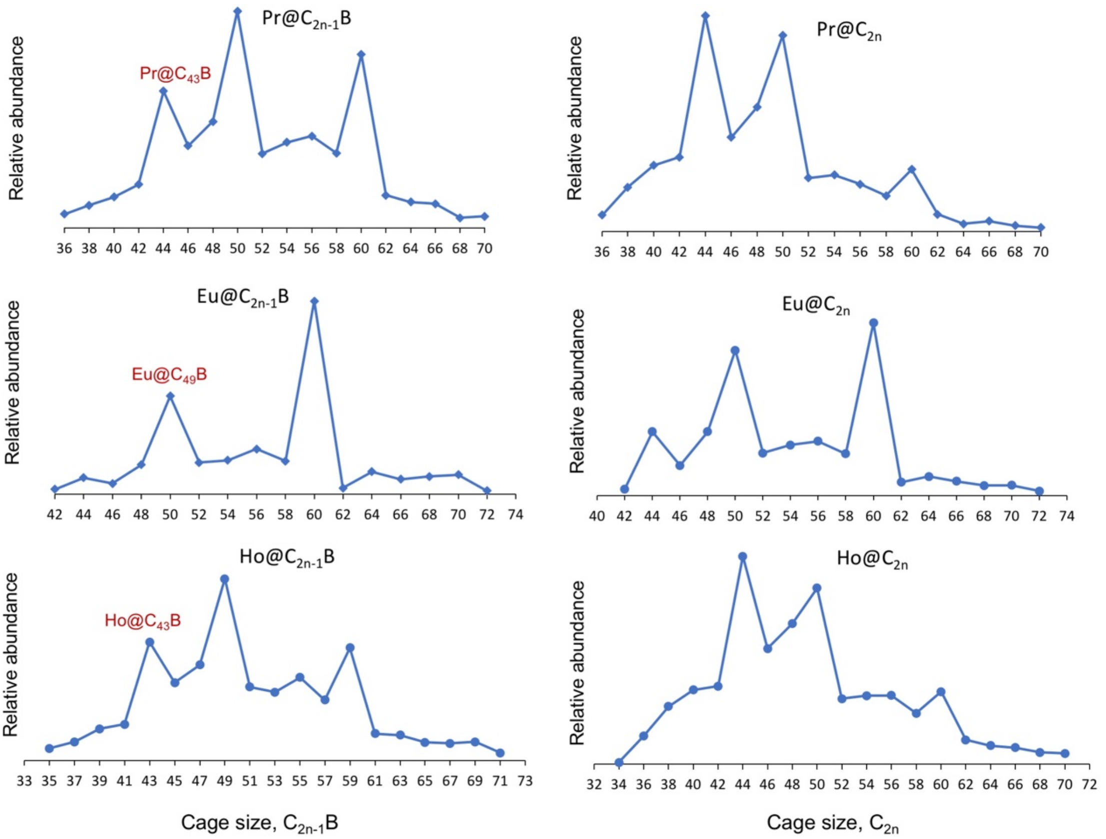

Figure S12. Relative abundances for boron-doped monometallofullerene families $M@C_{2n-1}B$ (M = Pr, Eu and Ho) obtained by FT-ICR mass spectrometry.

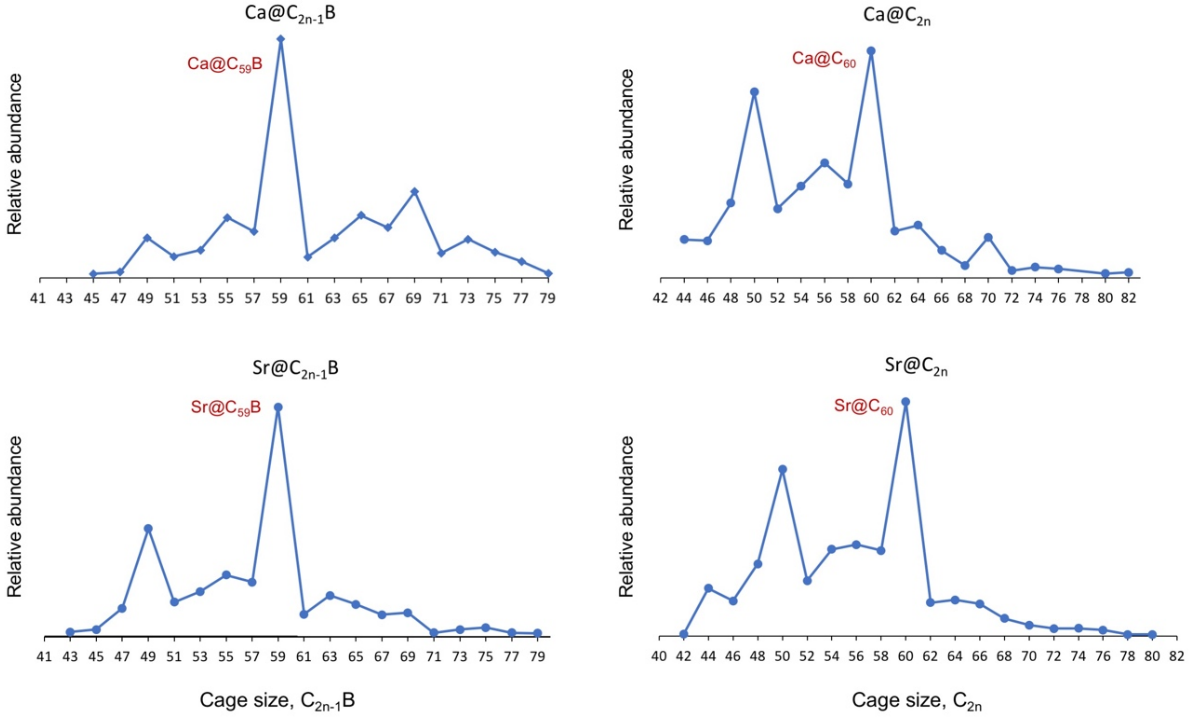

Figure S13. Relative abundances for boron-doped monometallofullerene families $M@C_{2n-1}B$ (M = Ca and Sr) obtained by FT-ICR mass spectrometry.

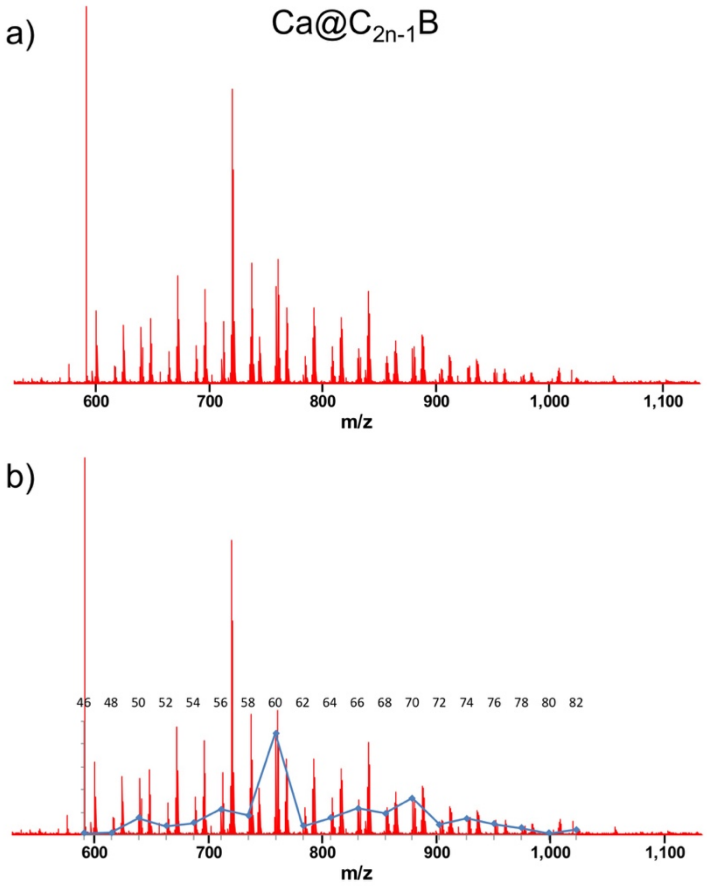

Figure S14. FT-ICR mass spectrum when using as starting materials graphite (99.9999%), boron powder (96%) and calcium oxide (a). The peaks for boron-doped monometallofullerene family $Ca@C_{2n-1}B$ are highlighted with the blue line (distribution peaks, see b).

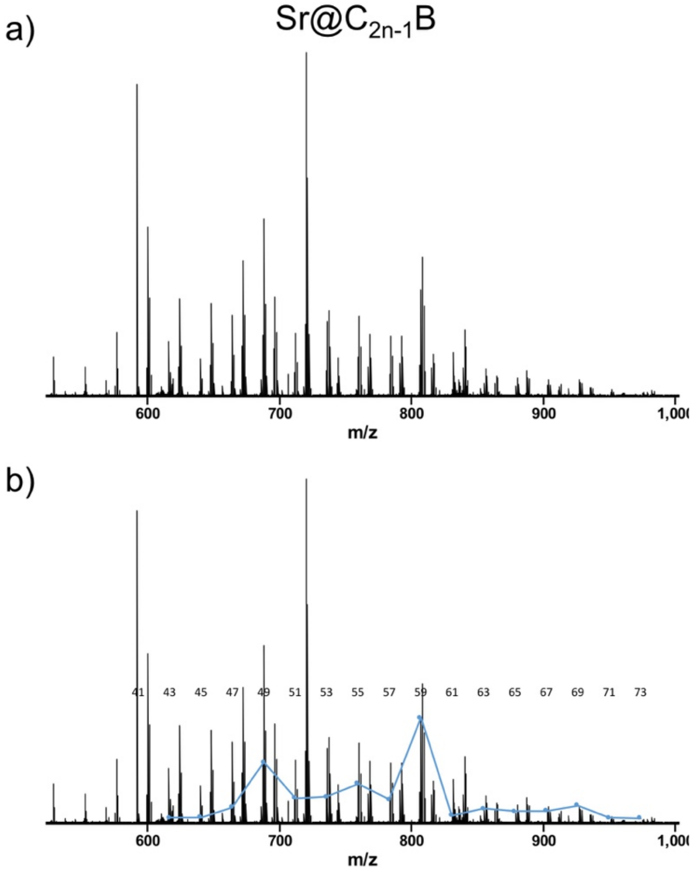

Figure S15. FT-ICR mass spectrum when using as starting materials graphite (99.9999%), boron powder (96%) and strontium oxide (a). The peaks for boron-doped monometallofullerene family Sr@C_{2n-1}B are highlighted with the blue line (distribution peaks, see b).

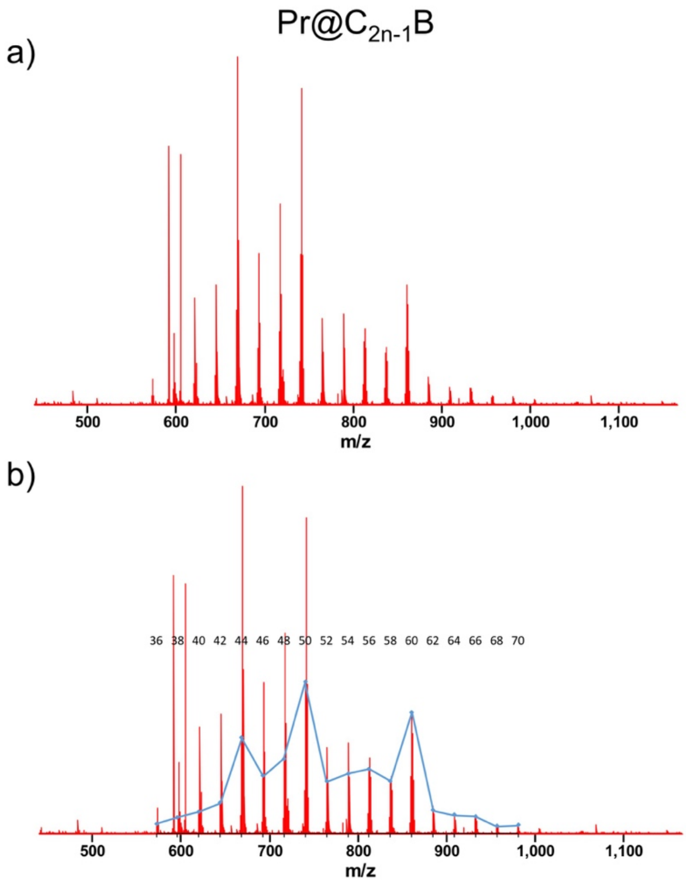

Figure S16. FT-ICR mass spectrum when using as starting materials graphite (99.9999%), boron powder (96%) and praseodymium oxide (a). The peaks for boron-doped monometallofullerene family Pr@C_{2n-1}B are highlighted with the blue line (distribution peaks, see b).

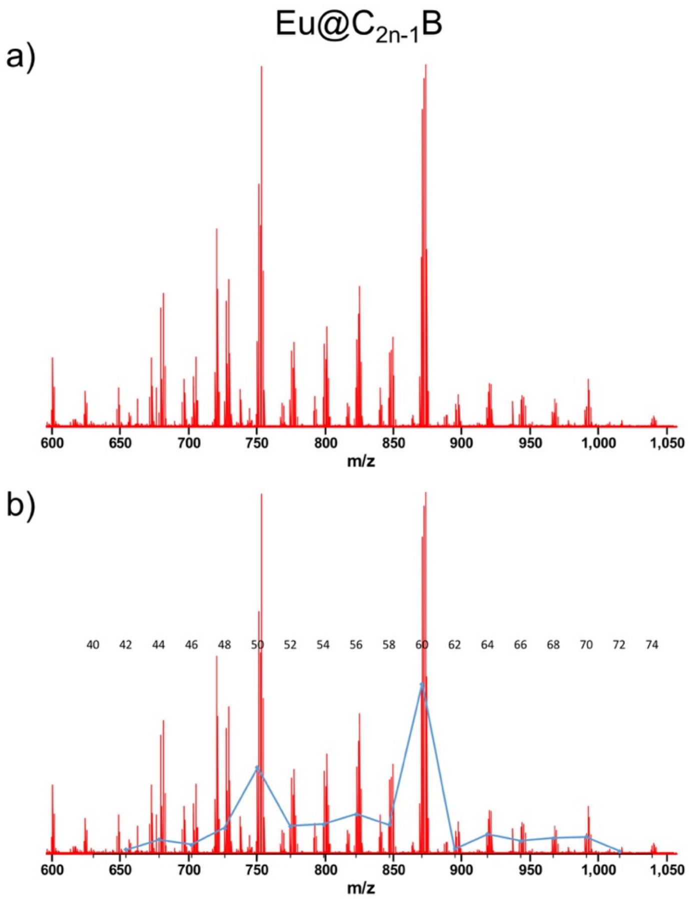

Figure S17. FT-ICR mass spectrum when using as starting materials graphite (99.9999%), boron powder (96%) and europium oxide (a). The peaks for boron-doped monometallofullerene family Eu@C_{2n-1}B are highlighted with the blue line (distribution peaks, see b).

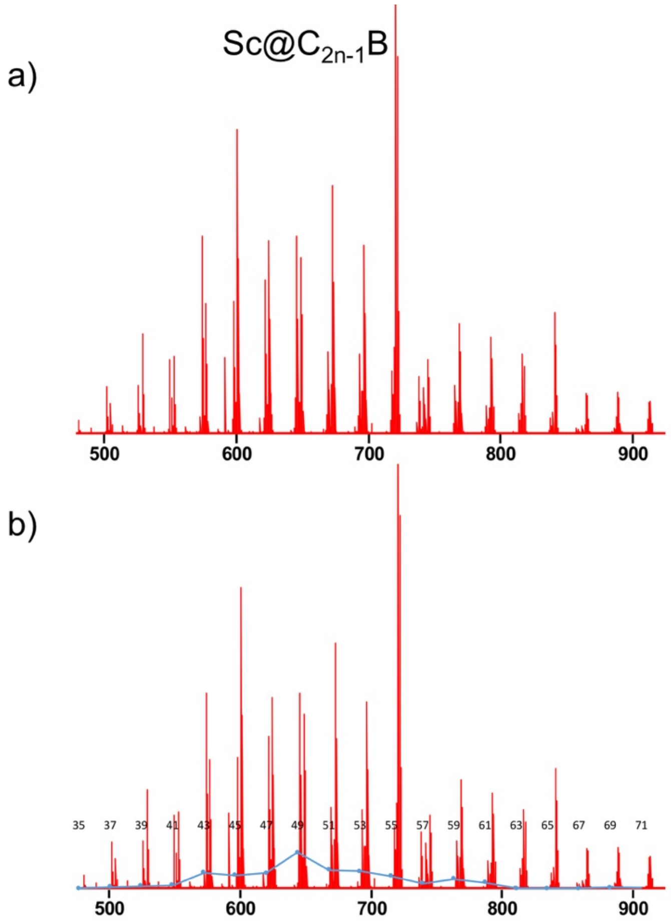

Figure S18. FT-ICR mass spectrum when using as starting materials graphite (99.9999%), boron powder (96%) and scandium oxide (a). The peaks for boron-doped monometallofullerene family Sc@C_{2n-1}B are highlighted with the blue line (distribution peaks, see b).

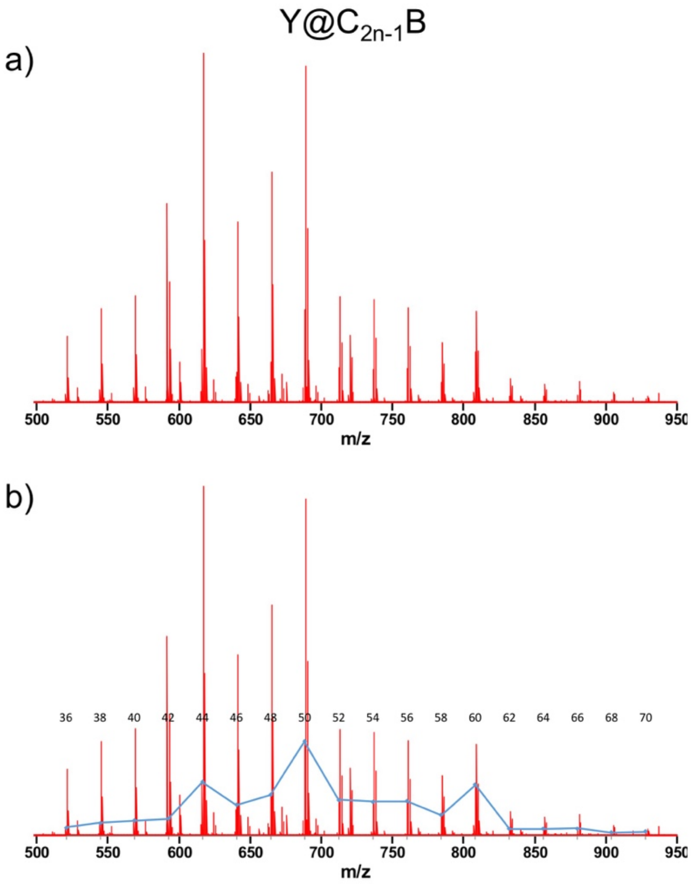

Figure S19. FT-ICR mass spectrum when using as starting materials graphite (99.9999%), boron powder (96%) and yttrium oxide (a). The peaks for boron-doped monometallofullerene family $Y@C_{2n-1}B$ are highlighted with the blue line (distribution peaks, see b).

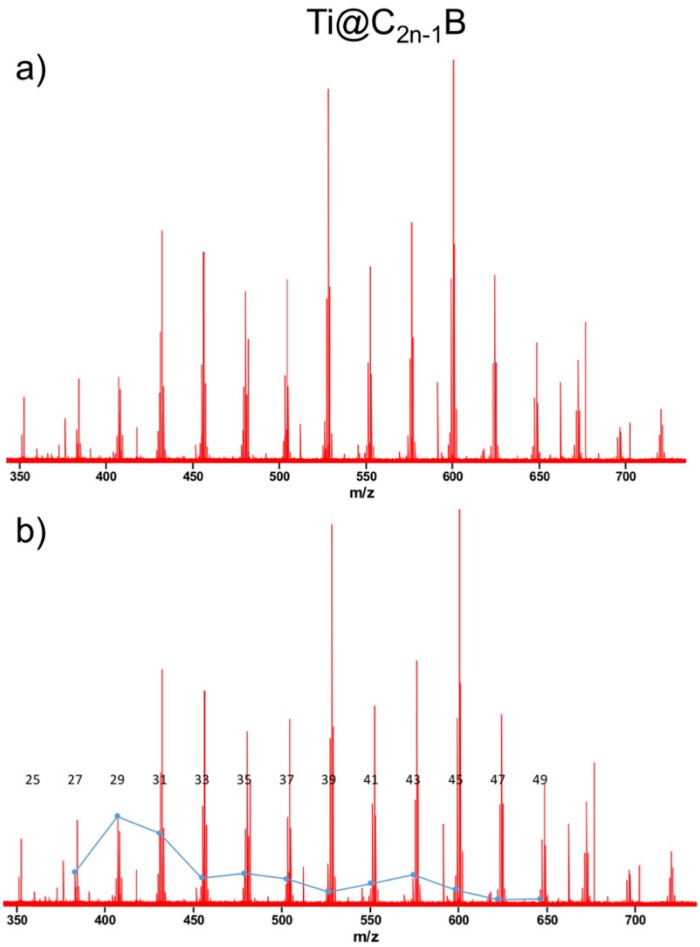

Figure S20. FT-ICR mass spectrum when using as starting materials graphite (99.9999%), boron powder (96%) and titanium oxide (a). The peaks for boron-doped monometallofullerene family $Ti@C_{2n-1}B$ are highlighted with the blue line (distribution peaks, see b).

Optimized xyz coordinates

$Sc_3N@C_{79}N$ (666 iso-1)

84

Sc	-0.176212	-0.661704	1.894422
Sc	-1.484799	0.145825	-1.222564
Sc	1.861581	0.965419	-0.503716
N	0.106048	0.126486	0.006268
C	-3.163817	2.407648	-0.163523
C	-3.650072	1.314645	-0.952492
C	-3.203687	1.199887	-2.330581
C	4.066470	0.015082	-1.372448
C	-2.962640	2.282403	1.254050
C	-2.354675	0.713284	3.020591
C	-3.211187	1.053692	1.924590
C	-3.743846	-0.029122	1.169327
C	-3.690153	-1.203270	-0.851997
C	-3.167297	-1.337674	-2.181590
C	-2.969994	-0.117223	-2.948548
C	-2.255282	-2.424266	-2.389646
C	-3.340024	-2.114322	0.208473
C	-1.568887	-3.546531	1.066455
C	-2.414708	-3.177605	-0.016176
C	-1.905397	-3.341489	-1.337416
C	-2.039049	-0.644075	3.411042
C	-2.473568	-1.721103	2.531067
C	-3.366544	-1.390111	1.452584
C	-1.598314	-2.844168	2.332486
C	0.607468	2.796914	-3.062785
C	0.130707	1.671302	-3.793129
C	-1.747273	-0.009238	-3.724292
C	-1.260055	1.336457	-3.627017
C	-0.238903	3.526304	-2.152164
C	-2.170972	3.340427	-0.666767
C	-1.608425	3.159438	-1.954016
C	-2.127889	2.073473	-2.753332
C	0.890920	3.765285	1.363986
C	0.018017	4.064077	0.275607
C	0.558706	3.969843	-1.043956
C	-1.374209	3.794067	0.443707
C	2.091202	-0.045551	3.550484
C	1.661838	-1.427818	3.568250
C	-0.284810	-2.916933	2.941813
C	3.228659	0.081323	2.672648
C	4.041605	1.191726	0.637344
C	3.455872	1.287341	1.953144
C	2.584874	2.388755	2.199032
C	3.505029	-2.432612	-0.038142
C	3.943761	-1.330637	0.745705
C	3.470499	-1.214796	2.083985
C	4.231658	-0.110230	0.045935
C	0.572651	-3.560218	1.961556
C	1.956690	-3.177546	1.775959
C	2.489071	-2.126293	2.603131
C	2.506856	-3.346398	0.476031
C	-0.220774	-3.989819	0.844266
C	-0.554974	-3.800909	-1.569733
C	0.315794	-4.082310	-0.476570
C	1.701580	-3.794438	-0.639533
C	3.802368	2.157279	-0.421296
C	2.799262	3.183476	-0.183430
C	2.241524	3.298580	1.132675
C	1.909749	3.509236	-1.260650
C	0.410614	3.130603	2.555510
C	-0.958471	2.758131	2.666603
C	-1.850466	3.131653	1.623408
C	-1.244607	1.546780	3.374083
C	1.452134	2.276123	3.078826
C	1.161402	1.036319	3.728995
C	-0.235920	0.708080	3.971859
C	3.807555	1.407698	-1.688006
C	2.356535	0.600213	-3.527061
C	2.838700	1.687058	-2.724665
C	1.937573	2.782194	-2.512022
C	3.308625	-2.311343	-1.453921
C	2.713566	-0.770118	-3.251215
C	3.548206	-1.091764	-2.141826
C	-3.936601	0.097232	-0.252857
C	-0.067180	-3.176490	-2.763718
C	1.606794	-1.611175	-3.627450
C	1.306428	-2.796623	-2.887881
C	2.185485	-3.158413	-1.825091
C	-1.108671	-2.322235	-3.274705
C	1.021792	0.604028	-4.075534
C	-0.810253	-1.096746	-3.919646
C	0.563215	-0.764103	-4.135865
C	-0.747869	-0.653471	4.098779
B	0.225437	-1.848629	3.912270

$Sc_3N@C_{79}N$ (665 iso-1)

84

Sc	-0.139840	-0.204652	2.003515
Sc	-1.514535	0.175256	-1.164598
Sc	1.995493	0.159405	-0.827079
N	0.148528	0.040188	-0.032563
C	-3.165174	2.408220	-0.173900
C	-3.656320	1.322791	-0.965267
C	-3.196612	1.208133	-2.341344
C	4.200138	0.029476	-1.383383
C	-2.976874	2.290608	1.252830
C	-2.372439	0.710693	3.035283
C	-3.208915	1.056265	1.919917
C	-3.740774	-0.029130	1.156813
C	-3.685681	-1.199737	-0.864320
C	-3.145769	-1.333702	-2.185160
C	-2.943676	-0.108596	-2.946589
C	-2.236011	-2.419688	-2.388704
C	-3.340787	-2.112942	0.198961
C	-1.564372	-3.532251	1.086583
C	-2.406935	-3.169673	-0.012825
C	-1.890126	-3.340506	-1.327509
C	-2.098208	-0.657805	3.421785
C	-2.506503	-1.709573	2.523081
C	-3.388987	-1.393641	1.441000
C	-1.608774	-2.813384	2.330804
C	0.609789	2.821146	-3.066846
C	0.132549	1.701015	-3.796835
C	-1.723663	0.002935	-3.717901
C	-1.250113	1.358077	-3.635641
C	-0.252719	3.553373	-2.161903
C	-2.176296	3.347324	-0.670295
C	-1.608120	3.175951	-1.967824
C	-2.124523	2.088506	-2.765128
C	0.873889	3.800575	1.362122
C	0.002591	4.082507	0.267492
C	0.543247	4.008709	-1.052222

C	-1.389333	3.796461	0.435015
C	2.025654	-0.017380	3.566132
C	0.223741	-1.798376	3.771885
C	1.586248	-1.409556	3.525613
C	-0.265718	-2.824984	2.896508
C	3.167531	0.104055	2.693306
C	3.967853	1.178983	0.655019
C	3.412005	1.313470	1.973828
C	2.565524	2.424721	2.207467
C	3.494646	-2.415674	-0.000787
C	3.932959	-1.309840	0.777330
C	3.433178	-1.185148	2.109095
C	4.273828	-0.097555	0.063122
C	0.585423	-3.546384	1.970252
C	1.948331	-3.174248	1.797389
C	2.450153	-2.109382	2.604700
C	2.518002	-3.348956	0.493558
C	-0.211803	-3.987455	0.865091
C	-0.541924	-3.791376	-1.550005
C	0.327764	-4.074021	-0.451329
C	1.720611	-3.788513	-0.615287
C	3.647734	2.098259	-0.397459
C	2.725245	3.183657	-0.172347
C	2.221024	3.350694	1.144781
C	1.891126	3.559189	-1.266402
C	0.383287	3.143443	2.543784
C	-0.990476	2.761278	2.666948
C	-1.879980	3.141576	1.624561
C	-1.268761	1.569627	3.426160
C	1.430783	2.304722	3.077776
C	1.125994	1.092791	3.782402
C	-0.247977	0.788415	4.111767
C	3.783809	1.410308	-1.663055
C	2.349470	0.629068	-3.494819
C	2.824907	1.720798	-2.702621
C	1.938834	2.828532	-2.513770
C	3.305083	-2.281760	-1.430065
C	2.724747	-0.741030	-3.210133
C	3.612574	-1.073299	-2.134532
C	-3.936582	0.099497	-0.268781
C	-0.051207	-3.160945	-2.742369
C	1.620275	-1.588143	-3.594811
C	1.324070	-2.778881	-2.865589
C	2.203673	-3.138682	-1.805285
C	-1.091839	-2.312868	-3.265701
C	1.025810	0.619919	-4.060593
C	-0.792109	-1.085227	-3.911636
C	0.579047	-0.747157	-4.124163
B	-0.760712	-0.684538	4.202392

$Sc_3N@C_{78}B_2$ (iso-1)

84

Sc	0.046952	-0.410350	1.959602
Sc	-1.482515	0.093296	-1.209376
Sc	1.864323	0.943300	-0.786569
N	0.132939	0.162045	-0.012931
C	-3.186537	2.396128	-0.157375
C	-3.660195	1.299418	-0.948928
C	-3.200065	1.185612	-2.318209
C	4.110731	0.030159	-1.341043
C	-2.982951	2.261238	1.259622
C	-2.346585	0.686550	3.000792
C	-3.218562	1.030163	1.911898
C	-3.761707	-0.054756	1.163174
C	-3.710931	-1.219203	-0.862718
C	-3.188820	-1.349570	-2.192846
C	-2.975187	-0.126457	-2.946785
C	-2.279665	-2.436867	-2.401129
C	-3.361813	-2.134138	0.195299
C	-1.608438	-3.599036	1.058191
C	-2.442733	-3.207104	-0.030005
C	-1.934019	-3.362021	-1.348714
C	-2.020101	-0.663554	3.365792
C	-2.485820	-1.754010	2.502020
C	-3.395036	-1.417677	1.442676
C	-1.634841	-2.878890	2.310080
C	0.617562	2.799378	-3.019190
C	0.142702	1.658862	-3.773373
C	-1.751064	-0.015142	-3.717781
C	-1.250851	1.326253	-3.606097
C	-0.241346	3.506504	-2.115933
C	-2.184920	3.317114	-0.651555
C	-1.613097	3.140329	-1.927433
C	-2.123102	2.056867	-2.734098
C	0.885170	3.758958	1.399426
C	0.014448	4.045781	0.308554
C	0.554819	3.962063	-1.003168
C	-1.381190	3.763104	0.471218
C	2.063125	-0.065350	3.594555
C	1.641954	-1.454034	3.611421
C	-0.307058	-2.920431	2.904395
C	3.186224	0.071153	2.699896
C	4.001278	1.173120	0.681757
C	3.431005	1.281701	1.996305
C	2.581764	2.391565	2.248593
C	3.486688	-2.436358	-0.010171
C	3.916485	-1.336379	0.775025
C	3.428399	-1.221557	2.105567
C	4.210663	-0.118794	0.084442
C	0.540914	-3.571946	1.953127
C	1.930184	-3.186070	1.784893
C	2.456824	-2.139222	2.625424
C	2.485489	-3.336144	0.492355
C	-0.258446	-4.025585	0.833095
C	-0.584272	-3.824174	-1.580653
C	0.278659	-4.096380	-0.481964
C	1.667112	-3.774727	-0.640766
C	3.804868	2.138712	-0.377076
C	2.790413	3.171132	-0.146250
C	2.238857	3.296532	1.173330
C	1.911234	3.528641	-1.235884
C	0.393108	3.121928	2.584306
C	-0.969510	2.743545	2.691645
C	-1.862142	3.110022	1.635882
C	-1.259962	1.533773	3.398205
C	1.436616	2.259149	3.109011
C	1.137124	1.028091	3.781223
C	-0.261549	0.706055	4.032808
C	3.936340	1.438060	-1.663239
C	2.388545	0.590054	-3.587761
C	1.972026	2.860361	-2.519271
C	3.279924	-2.284903	-1.427986
C	2.688307	-0.761166	-3.239686
C	3.540309	-1.075291	-2.108125
C	-3.953002	0.078082	-0.257126
C	-0.091599	-3.191132	-2.768513
C	1.580154	-1.619060	-3.617996
C	1.273390	-2.795513	-2.883579
C	2.148854	-3.141941	-1.805311
C	-1.129350	-2.328619	-3.274262
C	1.025169	0.593940	-4.092900
C	-0.822535	-1.104309	-3.924440
C	0.546250	-0.775358	-4.143112

C	-0.763648	-0.670472	4.096489
B	0.201305	-1.860519	3.897556
B	3.002354	1.787846	-2.835965

$Sc_3N@C_{77}B_3$ (iso-1)

84

Sc	-0.064353	-0.417639	1.927194
Sc	-1.620111	0.345306	-1.095578
Sc	1.831333	0.934879	-0.827790
N	0.112330	0.237691	0.008179
C	-3.228882	2.444781	-0.153773
C	-3.275000	1.199640	-2.431196
C	4.123300	0.020564	-1.334570
C	-2.975958	2.269242	1.251422
C	-2.324892	0.669632	2.978981
C	-3.204530	1.016007	1.889353
C	-3.759673	-0.078870	1.144971
C	-3.715761	-1.246217	-0.858369
C	-3.157958	-1.355515	-2.183791
C	-2.951188	-0.137057	-2.959867
C	-2.255656	-2.455340	-2.399690
C	-3.334437	-2.160090	0.189622
C	-1.575221	-3.612132	1.054708
C	-2.414216	-3.223896	-0.033799
C	-1.911207	-3.381197	-1.351744
C	-2.007374	-0.676641	3.371969
C	-2.455142	-1.766078	2.498154
C	-3.355389	-1.433010	1.433642
C	-1.600093	-2.889537	2.307306
C	0.628867	2.786376	-3.022833
C	0.161073	1.637678	-3.762543
C	-1.715032	-0.039467	-3.701124
C	-1.231635	1.308427	-3.603705
C	-0.240205	3.474594	-2.117117
C	-2.182496	3.308811	-0.647689
C	-1.619587	3.117819	-1.939887
C	-2.152011	2.052793	-2.786889
C	0.904489	3.749449	1.398408
C	0.029423	4.031215	0.310189
C	0.562824	3.932801	-1.002313
C	-1.362443	3.755579	0.471240
C	2.089001	-0.068303	3.591692
C	1.663141	-1.458435	3.599508
C	-0.278644	-2.937824	2.912342
C	3.218514	0.066836	2.713054
C	4.016262	1.165477	0.686987
C	3.456504	1.274628	2.002980
C	2.597439	2.380032	2.246516
C	3.498135	-2.446270	-0.006178
C	3.927307	-1.338666	0.783627
C	3.457045	-1.228431	2.115167
C	4.225488	-0.125259	0.089637
C	0.571910	-3.588525	1.963961
C	1.953516	-3.201075	1.791959
C	2.480873	-2.147148	2.631666
C	2.508340	-3.354587	0.496677
C	-0.230217	-4.037129	0.835673
C	-0.554090	-3.836976	-1.574661
C	0.309944	-4.117286	-0.481333
C	1.698051	-3.802973	-0.636542
C	3.807086	2.124291	-0.373426
C	2.800108	3.159139	-0.146366
C	2.256055	3.289368	1.172689
C	1.921344	3.515961	-1.238203
C	0.411384	3.111650	2.585444
C	-0.947983	2.733546	2.682460
C	-1.844448	3.105723	1.635941
C	-1.237101	1.513967	3.380143
C	1.454835	2.249864	3.111228
C	1.159214	1.017559	3.775321
C	-0.243586	0.691383	4.023723
C	3.942135	1.425787	-1.655947
C	2.410318	0.578216	-3.582637
C	1.986802	2.855077	-2.524780
C	3.300193	-2.302129	-1.421852
C	2.720134	-0.777082	-3.238432
C	3.558428	-1.086766	-2.104662
C	-4.045732	0.038194	-0.263971
C	-0.061429	-3.207093	-2.765611
C	1.609892	-1.635147	-3.612607
C	1.300589	-2.818031	-2.876364
C	2.178587	-3.166869	-1.802011
C	-1.107943	-2.352077	-3.276496
C	1.049704	0.575058	-4.079488
C	-0.796181	-1.126591	-3.906390
C	0.576695	-0.795360	-4.124096
C	-0.747484	-0.680335	4.102400
B	0.220813	-1.865035	3.889837
B	3.018065	1.780410	-2.837516
B	-3.872849	1.352853	-1.020274

$Rb@C_{59}B$

61

B	-3.636301	-0.707323	0.000000
C	-2.998744	-2.065698	0.000000
C	-2.245365	-2.484641	1.166335
C	-1.097140	-3.278392	0.726996
C	0.115228	-3.250818	1.427607
C	1.376171	-3.221114	0.700431
C	2.291446	-2.354678	1.428981
C	3.176253	-1.524205	0.729170
C	3.401329	-0.157387	1.178846
C	3.537981	0.687444	0.000000
C	3.013004	1.985870	0.000000
C	2.320412	2.488139	-1.179385
C	1.201796	3.303756	-0.728615
C	-0.013667	3.271361	-1.426572
C	-1.272573	3.240053	-0.698970
C	-2.198249	2.377618	-1.424571
C	-3.098495	1.545995	-0.739976
C	-3.399687	0.203468	-1.234422
C	-3.399687	0.203468	1.234422
C	-3.098495	1.545995	0.739976
C	-2.198249	2.377618	1.424571
C	-1.272573	3.240053	0.698970
C	-0.013667	3.271361	1.426572
C	1.201796	3.303756	0.728615
C	2.320412	2.488139	1.179385
C	2.183920	1.674759	2.312767
C	2.734866	0.324812	2.312684
C	1.817511	-0.538764	3.043539
C	1.591819	-1.851689	2.605870
C	0.247604	-2.408579	2.603635
C	-0.844235	-1.621787	3.015600
C	-2.113054	-1.659773	2.310992
C	-2.664297	-0.291776	2.322194
C	-1.719632	0.558157	3.030994
C	-1.504082	1.876366	2.602981
C	-0.156376	2.426157	2.604533
C	0.922166	1.642421	3.037818
C	0.694383	0.273842	3.484578

C	-0.603470	-0.255465	3.471909
C	-2.245365	-2.484641	-1.166335
C	-1.097140	-3.278392	-0.726996
C	0.115228	-3.250818	-1.427607
C	1.376171	-3.221114	-0.700431
C	2.291446	-2.354678	-1.428981
C	3.176253	-1.524205	-0.729170
C	3.401329	-0.157387	-1.178846
C	2.734866	0.324812	-2.312684
C	2.183920	1.674759	-2.312767
C	0.922166	1.642421	-3.037818
C	-0.156376	2.426157	-2.604533
C	-1.504082	1.876366	-2.602981
C	-1.719632	0.558157	-3.030994
C	-2.664297	-0.291776	-2.322194
C	-2.113054	-1.659773	-2.310992
C	-0.844235	-1.621787	-3.015600
C	0.247604	-2.408579	-2.603635
C	1.591819	-1.851689	-2.605870
C	1.817511	-0.538764	-3.043539
C	0.694383	0.273842	-3.484578
C	-0.603470	-0.255465	-3.471909
Rb	-0.313352	-0.073310	0.000000

Sr@C_{59}B
61

B	-3.620867	-0.691003	0.000000
C	-2.952774	-2.042371	0.000000
C	-2.185820	-2.469031	1.168277
C	-1.036946	-3.257916	0.722458
C	0.184430	-3.223342	1.431318
C	1.446407	-3.184497	0.699787
C	2.369415	-2.327803	1.430526
C	3.258464	-1.496567	0.725385
C	3.478221	-0.133174	1.175662
C	3.615724	0.712367	0.000000
C	3.085704	2.006426	0.000000
C	2.389318	2.508607	-1.175025
C	1.276891	3.330144	-0.723221
C	0.058632	3.298797	-1.433472
C	-1.198016	3.254776	-0.698590
C	-2.128288	2.392178	-1.420339
C	-3.049336	1.569696	-0.735596
C	-3.355581	0.224291	-1.231081
C	-3.355581	0.224291	1.231081
C	-3.049336	1.569696	0.735596
C	-2.128288	2.392178	1.420339
C	-1.198016	3.254776	0.698590
C	0.058632	3.298797	1.433472
C	1.276891	3.330144	0.723221
C	2.389318	2.508607	1.175025
C	2.256023	1.694751	2.305680
C	2.809817	0.351977	2.306750
C	1.885344	-0.509191	3.035001
C	1.669649	-1.822450	2.597818
C	0.317277	-2.382578	2.596591
C	-0.769640	-1.590328	3.020648
C	-2.043940	-1.632150	2.303528
C	-2.601494	-0.269849	2.315841
C	-1.646366	0.576972	3.028162
C	-1.431429	1.892905	2.592832
C	-0.081137	2.454051	2.599885
C	0.993865	1.660061	3.037275
C	0.770780	0.302753	3.489340
C	-0.537605	-0.236315	3.483501
C	-2.185820	-2.469031	-1.168277
C	-1.036946	-3.257916	-0.722458
C	0.184430	-3.223342	-1.431318
C	1.446407	-3.184497	-0.699787
C	2.369415	-2.327803	-1.430526
C	3.258464	-1.496567	-0.725385
C	3.478221	-0.133174	-1.175662
C	2.809817	0.351977	-2.306750
C	2.256023	1.694751	-2.305680
C	0.993865	1.660061	-3.037275
C	-0.081137	2.454051	-2.599885
C	-1.431429	1.892905	-2.592832
C	-1.646366	0.576972	-3.028162
C	-2.601494	-0.269849	-2.315841
C	-2.043940	-1.632150	-2.303528
C	-0.769640	-1.590328	-3.020648
C	0.317277	-2.382578	-2.596591
C	1.669649	-1.822450	-2.597818
C	1.885344	-0.509191	-3.035001
C	0.770780	0.302753	-3.489340
C	-0.537605	-0.236315	-3.483501
Sr	-0.865003	-0.264883	0.000000

Y@C_{59}B
61

B	-3.650604	-0.686569	0.000000
C	-2.912548	-2.038435	0.000000
C	-2.115001	-2.456993	1.157777
C	-0.964066	-3.251643	0.718916
C	0.249732	-3.243631	1.445330
C	1.507824	-3.202863	0.698939
C	2.424379	-2.344178	1.423976
C	3.311482	-1.514790	0.727303
C	3.540350	-0.150770	1.172692
C	3.683427	0.688408	0.000000
C	3.134640	1.987292	0.000000
C	2.434962	2.481880	-1.168952
C	1.315551	3.310367	-0.722487
C	0.107868	3.300246	-1.440331
C	-1.144638	3.257164	-0.700016
C	-2.068568	2.391591	-1.410726
C	-3.010463	1.583176	-0.737201
C	-3.351982	0.229725	-1.226001
C	-3.351982	0.229725	1.226001
C	-3.010463	1.583176	0.737201
C	-2.068568	2.391591	1.410726
C	-1.144638	3.257164	0.700016
C	0.107868	3.300246	1.440331
C	1.315551	3.310367	0.722487
C	2.434962	2.481880	1.168952
C	2.296549	1.674335	2.303615
C	2.859414	0.328285	2.304272
C	1.942090	-0.527304	3.021746
C	1.720910	-1.844901	2.601020
C	0.382934	-2.415809	2.610631
C	-0.711920	-1.606326	3.028839
C	-1.962666	-1.624769	2.295339
C	-2.540099	-0.255018	2.300701
C	-1.588596	0.577142	3.003800
C	-1.366623	1.906074	2.600662
C	-0.035506	2.464930	2.619066
C	1.047252	1.655816	3.045771
C	0.819565	0.297475	3.473655
C	-0.476557	-0.248821	3.462784
C	-2.115001	-2.456993	-1.157777
C	-0.964066	-3.251643	-0.718916
C	0.249732	-3.243631	-1.445330

C	1.507824	-3.202863	-0.698939
C	2.424379	-2.344178	-1.423976
C	3.311482	-1.514790	-0.727303
C	3.540350	-0.150770	-1.172692
C	2.859414	0.328285	-2.304272
C	2.296549	1.674335	-2.303615
C	1.047252	1.655816	-3.045771
C	-0.035506	2.464930	-2.619066
C	-1.366623	1.906074	-2.600662
C	-1.588596	0.577142	-3.003800
C	-2.540099	-0.255018	-2.300701
C	-1.962666	-1.624769	-2.295339
C	-0.711920	-1.606326	-3.028839
C	0.382934	-2.415809	-2.610631
C	1.720910	-1.844901	-2.601020
C	1.942090	-0.527304	-3.021746
C	0.819565	0.297475	-3.473655
C	-0.476557	-0.248821	-3.462784
Y	-1.284500	-0.201456	0.000000

Ti@C_{59}B

61

B	-3.717244	-0.665255	0.000000
C	-2.956007	-2.028988	0.000000
C	-2.117287	-2.415791	1.157964
C	-0.958251	-3.190460	0.721551
C	0.252688	-3.210245	1.442429
C	1.509066	-3.183938	0.699561
C	2.428873	-2.331015	1.424777
C	3.317311	-1.503395	0.728019
C	3.547753	-0.141801	1.174663
C	3.688781	0.697344	0.000000
C	3.156020	1.997724	0.000000
C	2.467609	2.499723	-1.174895
C	1.348904	3.315430	-0.729328
C	0.144662	3.306665	-1.438546
C	-1.110756	3.264883	-0.702542
C	-2.020411	2.410321	-1.421025
C	-2.930734	1.576742	-0.728291
C	-3.328809	0.240705	-1.217648
C	-3.328809	0.240705	1.217648
C	-2.930734	1.576742	0.728291
C	-2.020411	2.410321	1.421025
C	-1.110756	3.264883	0.702542
C	0.144662	3.306665	1.438546
C	1.348904	3.315430	0.729328
C	2.467609	2.499723	1.174895
C	2.324346	1.689623	2.310722
C	2.879352	0.340180	2.311537
C	1.961758	-0.512852	3.032098
C	1.733019	-1.829551	2.606648
C	0.393950	-2.384121	2.611206
C	-0.693942	-1.576034	3.027644
C	-1.941037	-1.584021	2.299337
C	-2.500596	-0.224277	2.299423
C	-1.560397	0.610947	3.011467
C	-1.340005	1.944029	2.626160
C	-0.002850	2.480101	2.626762
C	1.079027	1.675905	3.049794
C	0.842821	0.312849	3.480662
C	-0.452855	-0.217502	3.469593
C	-2.117287	-2.415791	-1.157964
C	-0.958251	-3.190460	-0.721551
C	0.252688	-3.210245	-1.442429
C	1.509066	-3.183938	-0.699561
C	2.428873	-2.331015	-1.424777
C	3.317311	-1.503395	-0.728019
C	3.547753	-0.141801	-1.174663
C	2.879352	0.340180	-2.311537
C	2.324346	1.689623	-2.310722
C	1.079027	1.675905	-3.049794
C	-0.002850	2.480101	-2.626762
C	-1.340005	1.944029	-2.626160
C	-1.560397	0.610947	-3.011467
C	-2.500596	-0.224277	-2.299423
C	-1.941037	-1.584021	-2.299337
C	-0.693942	-1.576034	-3.027644
C	0.393950	-2.384121	-2.611206
C	1.733019	-1.829551	-2.606648
C	1.961758	-0.512852	-3.032098
C	0.842821	0.312849	-3.480662
C	-0.452855	-0.217502	-3.469593
Ti	-1.613203	-0.404457	0.000000

Th@C_{59}B

61

B	-3.502928	-0.638742	0.000000
C	-2.802539	-2.019413	0.000000
C	-2.002059	-2.420942	1.174500
C	-0.823029	-3.140375	0.723805
C	0.413939	-3.138225	1.425901
C	1.660446	-3.127489	0.710432
C	2.574872	-2.261865	1.432057
C	3.448073	-1.421074	0.732586
C	3.674115	-0.062741	1.178623
C	3.797873	0.775363	0.000000
C	3.257107	2.070686	0.000000
C	2.572751	2.573453	-1.178433
C	1.453506	3.380268	-0.731194
C	0.242704	3.357597	-1.436253
C	-1.016093	3.324764	-0.711954
C	-1.909114	2.452834	-1.410201
C	-2.828506	1.603554	-0.735795
C	-3.181883	0.275200	-1.228514
C	-3.181883	0.275200	1.228514
C	-2.828506	1.603554	0.735795
C	-1.909114	2.452834	1.410201
C	-1.016093	3.324764	0.711954
C	0.242704	3.357597	1.436253
C	1.453506	3.380268	0.731194
C	2.572751	2.573453	1.178433
C	2.453764	1.765354	2.324346
C	3.015135	0.424241	2.324801
C	2.107732	-0.437791	3.049655
C	1.883973	-1.755099	2.618598
C	0.544961	-2.302783	2.605998
C	-0.550964	-1.516531	3.018655
C	-1.833895	-1.550002	2.320390
C	-2.394542	-0.214647	2.340489
C	-1.436491	0.642292	3.026773
C	-1.210418	1.960986	2.609814
C	0.114244	2.523628	2.625657
C	1.207486	1.739115	3.061154
C	0.983088	0.376062	3.495485
C	-0.313577	-0.164041	3.477265
C	-2.002059	-2.420942	-1.174500
C	-0.823029	-3.140375	-0.723805
C	0.413939	-3.138225	-1.425901
C	1.660446	-3.127489	-0.710432
C	2.574872	-2.261865	-1.432057
C	3.448073	-1.421074	-0.732586
C	3.674115	-0.062741	-1.178623

C	3.015135	0.424241	-2.324801
C	2.453764	1.765354	-2.324346
C	1.207486	1.739115	-3.061154
C	0.114244	2.523628	-2.625657
C	-1.210418	1.960986	-2.609814
C	-1.436491	0.642292	-3.026773
C	-2.394542	-0.214647	-2.340489
C	-1.833895	-1.550002	-2.320390
C	-0.550964	-1.516531	-3.018655
C	0.544961	-2.302783	-2.605998
C	1.883973	-1.755099	-2.618598
C	2.107732	-0.437791	-3.049655
C	0.983088	0.376062	-3.495485
C	-0.313577	-0.164041	-3.477265
Th	-1.004208	-0.323240	0.000000

Y@C_{49}B
51

C	0.051707	1.341218	3.329438
C	0.065342	2.456931	2.392072
C	1.327056	2.528945	1.660240
C	2.191633	1.406317	2.085932
B	1.447115	0.646637	3.251584
C	-1.204148	0.626338	3.104240
C	-1.968046	1.342613	2.060495
C	-1.177685	2.503904	1.657938
C	-1.195399	2.970325	0.316273
C	0.037262	3.371929	-0.289822
C	1.280034	2.972324	0.309367
C	2.044017	2.262139	-0.720316
C	2.762396	1.104580	-0.360575
C	2.816405	0.660989	1.030294
C	2.788861	-0.812120	1.013346
C	2.084915	-1.556104	2.006829
C	1.374764	-0.864676	3.096973
C	0.080910	-1.530935	3.185727
C	-1.176027	-0.814430	3.039763
C	-1.954597	-1.533004	2.012337
C	-2.685335	-0.816099	1.035562
C	-2.684780	0.636276	1.049850
C	-2.695932	1.106972	-0.329144
C	-1.979383	2.264225	-0.695860
C	-1.216823	2.285504	-1.943943
C	0.027748	2.948822	-1.675846
C	1.266634	2.282940	-1.957897
C	1.260667	1.145104	-2.800142
C	2.032176	-0.048039	-2.437887
C	2.747091	-0.065869	-1.233063
C	2.755102	-1.252798	-0.382475
C	2.043846	-2.401839	-0.770182
C	1.276178	-3.141040	0.234281
C	1.292322	-2.716980	1.582238
C	0.054964	-2.676666	2.310571
C	-1.194433	-2.711801	1.601927
C	-1.207084	-3.139601	0.253088
C	-1.987368	-2.401738	-0.745194
C	-2.701702	-1.257271	-0.358345
C	-2.706627	-0.063933	-1.202737
C	-1.992106	-0.046151	-2.409609
C	-1.220926	1.145747	-2.781490
C	0.015960	0.690608	-3.335674
C	0.015190	-0.763091	-3.355463
C	1.256863	-1.229424	-2.827833
C	1.262580	-2.382123	-2.005564
C	0.022490	-3.046735	-1.736591
C	0.029718	-3.515801	-0.365460
C	-1.219250	-2.381658	-1.990608
C	-1.222342	-1.226042	-2.806831
Y	0.201414	0.237801	1.171275

Th@C_{49}B
51

C	0.059560	1.293391	3.438327
C	0.073513	2.391857	2.491797
C	1.326686	2.469964	1.762303
C	2.193426	1.364552	2.191916
B	1.459779	0.611554	3.365712
C	-1.208361	0.596485	3.237256
C	-1.966554	1.308784	2.189014
C	-1.168532	2.453807	1.770442
C	-1.187847	2.924819	0.427909
C	0.037833	3.331083	-0.184509
C	1.277700	2.924069	0.413289
C	2.036915	2.221583	-0.616930
C	2.772960	1.071445	-0.257963
C	2.841248	0.627582	1.145130
C	2.831884	-0.829327	1.137878
C	2.124226	-1.579647	2.153918
C	1.416241	-0.901028	3.252858
C	0.091313	-1.549714	3.337472
C	-1.190803	-0.845380	3.203078
C	-1.960064	-1.550099	2.154384
C	-2.695379	-0.829047	1.161155
C	-2.695715	0.607714	1.176331
C	-2.698755	1.075210	-0.217434
C	-1.970098	2.223071	-0.585245
C	-1.215045	2.251731	-1.839052
C	0.025301	2.931730	-1.579016
C	1.260008	2.247500	-1.853922
C	1.254304	1.110016	-2.697783
C	2.026463	-0.074900	-2.334542
C	2.764183	-0.086345	-1.123155
C	2.762687	-1.262469	-0.268731
C	2.037305	-2.403716	-0.663827
C	1.277134	-3.139827	0.342980
C	1.305149	-2.702868	1.692474
C	0.065564	-2.651677	2.416733
C	-1.186029	-2.698947	1.712062
C	-1.196188	-3.138487	0.361782
C	-1.976050	-2.409868	-0.636655
C	-2.699379	-1.268620	-0.242350
C	-2.722470	-0.085638	-1.087366
C	-1.988826	-0.068993	-2.301442
C	-1.225787	1.113537	-2.679238
C	0.012814	0.661915	-3.269929
C	0.013484	-0.779951	-3.280001
C	1.259723	-1.245850	-2.727812
C	1.260581	-2.398446	-1.902683
C	0.026657	-3.082530	-1.640552
C	0.035534	-3.526902	-0.257237
C	-1.213030	-2.400473	-1.885802
C	-1.220844	-1.242624	-2.701055
Th	0.172758	-0.046511	1.185577

Th@C_{43}B
45

C	0.532522	3.453887	0.524647
C	-0.633571	3.539231	-0.365013
C	-0.231536	2.960169	-1.620875
C	1.186478	2.615554	-1.553627

C	1.648736	2.866089	-0.191006
C	0.133814	2.910290	1.808137
C	-1.311079	2.638248	1.806167
B	-1.874434	3.022057	0.392260
C	-2.627129	1.904227	-0.329978
C	-2.169317	1.362370	-1.647671
C	-0.948535	1.864043	-2.263443
C	0.084073	0.879191	-2.683124
C	1.402048	1.349712	-2.197070
C	2.328423	0.431496	-1.632486
C	2.937272	0.761381	-0.347962
C	2.450733	1.861663	0.440423
C	2.052847	1.312830	1.756452
C	0.853077	1.799819	2.391897
C	-0.159672	0.816107	2.822296
C	-1.486267	1.326882	2.383695
C	-2.390207	0.401289	1.755969
C	-2.987333	0.742526	0.459246
C	-2.946700	-0.439102	-0.347270
C	-2.384503	-0.086406	-1.630395
C	-1.465412	-1.017220	-2.203713
C	-0.159715	-0.510366	-2.671672
C	0.865875	-1.471806	-2.249240
C	2.059449	-0.997991	-1.640446
C	2.479383	-1.544373	-0.333539
C	2.947355	-0.428631	0.447810
C	2.351214	-0.110489	1.731307
C	1.436734	-1.051450	2.289470
C	0.121720	-0.566607	2.770748
C	-0.890131	-1.522870	2.324477
C	-2.082256	-1.031831	1.726706
C	-2.483177	-1.565955	0.411573
C	-1.707412	-2.557817	-0.243223
C	-1.256222	-2.281414	-1.591637
C	0.181828	-2.585739	-1.649852
C	0.590198	-3.125967	-0.420311
C	1.703291	-2.552408	0.300516
C	1.240688	-2.305748	1.653586
C	-0.194219	-2.621702	1.701828
C	-0.591827	-3.144020	0.463674
Th	-0.197802	0.966929	0.080623

Th@C$_{35}$B(14)
37

C	0.376082	1.624514	2.507284
C	0.543364	2.702934	1.523358
C	1.700719	2.365612	0.770408
C	2.287101	1.112900	1.224985
C	1.417950	0.614276	2.319793
C	-1.030897	1.236532	2.543167
C	-1.810272	1.943070	1.488001
B	-0.833745	2.910062	0.786183
C	-0.801686	2.821412	-0.779666
C	0.493720	2.651982	-1.445274
C	1.685942	2.367008	-0.688757
C	2.266156	1.127410	-1.147824
C	2.608622	0.312916	0.032557
C	2.240901	-1.082326	0.018695
C	1.572164	-1.643214	1.222733
C	1.062789	-0.786611	2.321307
C	-0.340032	-1.142220	2.511298
C	-1.366422	-0.127502	2.552526
C	-2.317591	-0.369719	1.446673
C	-2.544390	0.915933	0.766775
C	-2.524869	0.906811	-0.699893
C	-1.723367	1.925079	-1.437063
C	-1.009838	1.237477	-2.531875
C	0.374310	1.614623	-2.472771
C	1.417394	0.618152	-2.263321
C	1.066639	-0.762563	-2.252075
C	1.575943	-1.620783	-1.165894
C	0.443798	-2.409654	-0.687094
C	0.462833	-2.439020	0.757859
C	-0.720972	-2.091359	1.474027
C	-1.907531	-1.575354	0.764183
C	-1.911356	-1.573609	-0.691964
C	-2.312328	-0.360795	-1.371112
C	-1.346484	-0.130445	-2.498266
C	-0.347116	-1.126275	-2.447182
C	-0.732471	-2.096658	-1.402013
Th	-0.005921	0.327778	0.126009

Th@C$_{35}$B(15)
37

C	0.624222	-2.714425	0.928194
C	1.843518	-2.226226	0.252948
C	1.576091	-2.034757	-1.136186
C	0.152679	-2.346668	-1.380392
C	-0.429621	-2.744881	-0.082182
B	0.482399	-1.874874	2.241162
C	1.661272	-0.846174	2.231233
C	2.450354	-1.132282	1.015860
C	2.755441	0.090520	0.346103
C	2.497052	0.269454	-1.059598
C	1.902354	-0.802970	-1.807998
C	0.697424	-0.301576	-2.467694
C	-0.389707	-1.272767	-2.206252
C	-1.690110	-0.755870	-1.870232
C	-2.396853	-1.340873	-0.735172
C	-1.744815	-2.195090	0.245047
C	-2.007980	-1.602472	1.574201
C	-0.921742	-1.263354	2.481284
C	-1.034375	0.152784	2.798748
C	0.078495	1.090593	2.536161
C	1.423149	0.594172	2.245866
C	2.118854	1.197029	1.090485
C	1.454907	2.051234	0.111770
C	1.680287	1.469994	-1.231798
C	0.557376	1.113309	-2.105593
C	-0.760234	1.609009	-1.767741
C	-1.897064	0.694712	-1.826128
C	-2.756394	0.988708	-0.670681
C	-3.067830	-0.231260	-0.012028
C	-2.829978	-0.397941	1.379251
C	-2.235255	0.674561	2.127750
C	-1.893959	1.883636	1.462994
C	-0.455415	2.158203	1.704641
C	0.136629	2.536060	0.424959
C	-0.915940	2.462756	-0.593253
C	-2.160490	2.063029	0.078537
Th	0.057533	-0.178943	0.146045

Ti@C$_{43}$B
45

C	0.555098	3.328933	0.505250
C	-0.632665	3.415794	-0.394172
C	-0.221865	2.755634	-1.626163
C	1.181180	2.388497	-1.547364
C	1.657250	2.665370	-0.205874
C	0.147310	2.750893	1.786495
C	-1.297377	2.457704	1.751855

B	-1.885221	2.866926	0.360678
C	-2.577158	1.679192	-0.328920
C	-2.115588	1.111336	-1.623637
C	-0.913891	1.611073	-2.216780
C	0.109542	0.643581	-2.657649
C	1.416386	1.124102	-2.193013
C	2.364637	0.229264	-1.656021
C	2.984283	0.568699	-0.386017
C	2.482706	1.660527	0.398110
C	2.062273	1.098936	1.695092
C	0.849707	1.584593	2.286983
C	-0.153265	0.600739	2.708134
C	-1.460486	1.107689	2.279895
C	-2.381270	0.187126	1.699926
C	-3.018687	0.526773	0.432785
C	-2.974780	-0.653022	-0.373727
C	-2.381458	-0.312309	-1.645603
C	-1.452507	-1.228792	-2.225025
C	-0.145070	-0.731688	-2.703472
C	0.887226	-1.671266	-2.272004
C	2.081673	-1.201725	-1.667231
C	2.501076	-1.739300	-0.360976
C	2.971333	-0.623476	0.415988
C	2.366035	-0.310793	1.692531
C	1.446411	-1.247330	2.259973
C	0.129228	-0.770855	2.733802
C	-0.887336	-1.717489	2.297294
C	-2.080787	-1.242100	1.696140
C	-2.498353	-1.777637	0.389181
C	-1.706537	-2.754558	-0.266216
C	-1.246656	-2.485130	-1.611435
C	0.198555	-2.779808	-1.662815
C	0.603343	-3.305582	-0.437644
C	1.717748	-2.733391	0.282263
C	1.255911	-2.496571	1.631862
C	-0.186480	-2.813909	1.678617
C	-0.583620	-3.322919	0.444831
Ti	-0.226847	1.424230	0.141688

Ti@C_{29}B(3)
31

C	-2.238395	-0.138682	0.672886
C	-1.623795	0.065913	2.002511
C	-0.937272	-1.105247	2.418908
C	-1.052236	-2.181424	1.416430
B	-1.856713	-1.582368	0.194214
C	-1.901475	1.006375	-0.138447
C	-0.913045	1.833733	0.635481
C	-0.652660	1.130093	1.873840
C	0.702702	0.588610	2.160027
C	0.488800	-0.795007	2.584710
C	1.234962	-1.854211	1.990402
C	0.250197	-2.690737	1.221554
C	0.808082	-2.856366	-0.103258
C	0.116837	-2.334959	-1.255503
C	-1.191293	-1.669930	-1.209183
C	-1.045412	-0.443726	-2.040191
C	-1.421467	0.894706	-1.527622
C	-0.201057	1.752013	-1.605354
C	0.162986	2.228900	-0.243420
C	1.509938	1.755497	0.060612
C	1.783432	0.893518	1.242846
C	2.611602	-0.211940	0.764648
C	2.321155	-1.567749	1.113719
C	2.176703	-2.341752	-0.127371
C	2.351317	-1.472288	-1.251261
C	1.085942	-1.518387	-1.994153
C	0.400482	-0.358402	-2.471312
C	0.922121	0.965816	-2.129033
C	2.011134	1.030079	-1.141014
C	2.670186	-0.160341	-0.710669
Ti	0.080619	0.126811	-0.358380

Ti@C_{27}B
29

C	-0.205396	-1.241186	2.359935
C	-0.449433	0.191371	2.580063
C	0.764494	0.939029	2.330688
C	1.798473	-0.036672	2.087799
C	1.201246	-1.418169	2.119794
C	-1.180183	-1.686407	1.405859
C	-1.960112	-0.463040	0.961428
C	-1.480973	0.694040	1.695664
C	-1.007407	1.912424	1.007759
C	0.448070	2.024876	1.396045
C	1.273444	2.168530	0.178588
C	2.284352	1.146279	-0.057212
C	2.600194	0.092473	0.902549
C	2.571969	-1.183342	0.223247
C	1.638295	-2.086980	0.922226
C	0.670460	-2.542800	-0.041708
C	-0.731357	-2.354961	0.218579
C	-1.309698	-1.629641	-0.922166
C	-2.017204	-0.442271	-0.489034
C	-1.565650	0.727733	-1.268372
B	-1.125667	2.047925	-0.548804
C	0.372862	2.222818	-1.007559
C	0.764212	1.090597	-1.893776
C	1.967053	0.473980	-1.350170
C	2.101784	-0.962250	-1.126244
C	0.960713	-1.812535	-1.325638
C	-0.256686	-1.224137	-1.827930
C	-0.407275	0.211696	-2.088077
Ti	0.141224	0.401353	-0.000728
